# The study on the identification of cross-boundary microbiome enterotypes between high-altitude and coastal populations and their predictive value

**DOI:** 10.1186/s12866-025-04578-0

**Published:** 2026-01-29

**Authors:** Jiawei Zhang, Jiaxin Deng, Bingfeng He, Han Wang, Dezheng Lin, Juan Li, Qinghua Zhong, Yongcheng Chen, Sen Liao, Junhao Wang, Yuying Wang, Mingli Su, Xuefeng Guo

**Affiliations:** 1 Department of General Surgery (Endoscopic Surgery), The Sixth Affiliated Hospital, Sun Yat-sen University, Guangzhou, China; 2 Guangdong Provincial Key Laboratory of Colorectal and Pelvic Floor Diseases, The Sixth Affiliated Hospital, Sun Yat-sen University, Guangzhou, China; 3 Biomedical Innovation Center, The Sixth Affiliated Hospital, Sun Yat-sen University, Guangzhou, China; 4 The Medical College of Xizang Minzu University, Xianyang, Shaanxi China

**Keywords:** Colorectal adenoma, Gut microbiota, High-altitude, Enterotype

## Abstract

**Objective:**

To investigate the differences in gut microbiome composition among multi-center populations from coastal and high-altitude regions of China and their association with colorectal adenoma (CRA).

**Methods and analysis:**

Metagenomic sequencing was performed on stool samples collected from 295 participants. Diversity, principal component, and linear discriminant analyses were conducted to assess microbial composition and functional differences related to geography and disease status.

**Results:**

In high-altitude populations, bacterial enterotypes were predominantly Prevotella, fungal enterotypes Saccharomyces, and archaeal enterotypes Methanobrevibacter, differing from those in coastal populations. Combining bacterial, fungal, and archaeal features improved classification accuracy between high-altitude and coastal populations (AUC = 0.84) and between high-altitude and coastal adenoma patients (AUC = 0.85). Specific enterotypes were observed to correlate significantly with metabolic pathways in high-altitude populations.

**Conclusion:**

Significant differences in gut microbiome enterotypes exist across geographic populations, with specific enterotypes in high-altitude populations potentially associated with a lower prevalence of CRA. These findings provide new insights into the gut microbiome–geography relationship and support microbiome-based diagnostic and therapeutic strategies.

**Supplementary Information:**

The online version contains supplementary material available at 10.1186/s12866-025-04578-0.

## Key messages

What is already known on this topic: The gut microbiome plays a crucial role in the progression of adenomas to colorectal cancer, but how different geographic environments impact the gut microbiome remains unclear.

What this study adds: This study reveals significant differences in gut microbiome composition between high-altitude and coastal populations, highlighting potential protective effects in high-altitude areas.

How this study might affect research, practice or policy: Emphasizes the importance of geographic factors in developing personalized prevention and treatment strategies for CRA, underscoring the need for further longitudinal cohort studies to validate these findings and explore underlying mechanisms.

**Supplementary Information:**

The online version contains supplementary material available at 10.1186/s12866-025-04578-0.

## Introduction

Colorectal adenoma (CRA) is a common precursor lesion of colorectal cancer globally, with the latest epidemiological surveys showing a rising incidence of early-onset CRA, which is associated with poor prognosis [1]. CRA account for 85%-90% of all colorectal cancer precursor lesions [2], and most colorectal cancers develop through the adenoma-carcinoma sequence [3]. Early detection and intervention are crucial for preventing adenoma progression to cancer, but many patients are diagnosed at advanced stages, making early screening and preventive measures essential [4, 5]. The risk factors for CRA include diet, geography, altitude, and gut microbiota [6–8].

The gut microbiota has been recognized as a key factor in the development of colorectal cancer and may be involved in the formation of adenomas and their progression to colorectal cancer [9]. The human gut microbiome consists of microorganisms from multiple kingdoms, such as bacteria, viruses, protozoa, and fungi, all of which are crucial for health [10]. Increasing evidence suggests that non-bacterial microorganisms, particularly fungi, are associated with cancer development, with fungal species within tumors correlating significantly with clinical outcomes [11]. For example, Hoarau et al. [12] found a positive correlation between fungal and bacterial species in Crohn’s disease patients. Additionally, altitude is an important factor influencing the gut microbiota. The Tibetan Plateau, with altitudes exceeding 4000 m, has significantly lower oxygen pressure than lowland areas, a unique environment oxygen tension, and traditional diet composition that may lead to changes in the gut microbiota structure and play an important role in adapting to the hypoxic high-altitude environment [13, 14]. Different genetic and environmental factors may determine the composition and function of the gut microbiome in high-altitude populations [15–17]. As altitude increases, the gut microbiomes of high-altitude residents show significant diversity and unique species composition [18, 19]. However, epidemiological studies indicate that the incidence of colorectal cancer is significantly lower in high-altitude regions compared to other areas, while coastal regions exhibit higher rates of incidence and mortality [20]. Multiple factors including lifestyle, environmental exposure and dietary habits may contribute to these cancer incidence differences. This observation raises the possibility of gut microbiota variations between populations and implies a potential complex relationship between gut microbiota and colorectal cancer incidence. The concept of enterotypes can simplify these analyses. Enterotype classification based on the gut microbiota helps reveal the symbiotic relationship between the host and microorganisms. In 2011, Arumugam et al. [21] introduced the concept of enterotypes through studies on the human microbiome genome, identifying three major enterotypes: Bacteroides, Prevotella, and Ruminococcus. The advantage of enterotype classification is that it represents the microbial profiles of many individuals through a few typical patterns, which can be combined with regional characteristics to assess disease risks and drug responses. Specific enterotypes have been linked to diseases such as colorectal cancer and diabetes [22, 23], and increasing evidence suggests that enterotypes play an important role in various pathophysiological processes [22, 24]. Yang et al. [25] found that different enterotypes in CRA patients exhibited distinct dysbiotic features in their gut microbiomes. However, research on enterotypes in high-altitude populations is limited, particularly concerning the enterotypes of high-altitude CRA patients. Therefore, exploring whether low-abundance microbiome enterotypes, particularly those involving fungi and archaea, are associated with CRA occurrence in different regional populations is of great significance. Based on cross-boundary enterotype classification, grouping patients from different regions will help achieve personalized clinical diagnosis and treatment based on the microbiome.

This study aims to investigate the enterotype characteristics of bacteria, fungi, and archaea in the gut microbiota of coastal and high-altitude populations and their role in predicting CRA. We will collect stool samples from multi-center populations in coastal and high-altitude regions, stratifying them according to colonoscopy and histopathological results. We will comprehensively analyze the compositional and functional differences of these three microbiome types across different enterotypes via diversity and principal component analysis, and construct enterotype-based predictive models for CRA in populations from different regions. This study aims to provide new biomarkers for early screening of CRA in different geographic populations and offer theoretical support for developing region-specific preventive and diagnostic strategies, thereby expanding the potential application of enterotypes in disease prediction and health management.

## Materials and methods

### Human subjects and sample collection

Microbiome analysis samples were obtained by collecting stool from coastal and high-altitude residents. The inclusion criteria were: (1) aged 18–75 years, regularly attending outpatient examinations; (2) no history of tumors. The exclusion criteria were: (1) use of antibiotics (antibacterial or antifungal medications) within 3 months prior to colonoscopy, use of microbiological agents (such as probiotics and prebiotics) within 1 month, or any medications that could affect the gut microbiota or host immunity, such as hormones and immunosuppressants; (2) family history of familial adenomatous polyposis; (3) family history of inflammatory bowel disease; (4) acute infectious diseases; (5) history of gastrointestinal surgery; (6) incomplete colonoscopy or presence of high-risk factors for bleeding or perforation; (7) co-existing severe cardiovascular or pulmonary diseases. Written informed consent was obtained from all participants prior to the study. Sample collection was carried out at multiple centers, with high-altitude samples obtained from large hospitals in Tibet and Qinghai, and coastal samples from large hospitals in Guangdong and Fujian. Recruitment took place from January 1, 2019, to January 1, 2024. Stool samples were collected before colonoscopy and strictly followed established procedures. The samples were immediately transferred into centrifuge tubes and stored at -80 °C until sequencing. Participants were grouped according to the colonoscopy report. The diagnosis of adenomas was based on a comprehensive evaluation of endoscopic findings, clinical data, and histopathological results. Specifically, the Coastal Healthy Population (CHP) included 78 individuals, the High-altitude Healthy Population (HHP) included 68 individuals, the Coastal Adenoma Population (CAP) included 106 individuals, and the High-altitude Adenoma Population (HAP) included 43 individuals. To address potential confounding between altitude and disease status, we adopted a two-tiered analytical design. The primary comparison between coastal populations (CP) and high-altitude populations (HP) included all participants (*n* = 295) to capture comprehensive population-level microbial patterns inherently associated with geographic origin. To disentangle the independent effects of disease, we conducted separate subgroup analyses: HAP versus CAP, and HCP versus HAP. This design ensures that origin-driven microbial signals are not obscured by disease-related variations, as the subgroup analyses isolate disease-specific impacts while the primary analysis retains the integrity of population-level comparisons. This study protocol was approved by the Ethics Committee of the Sixth Affiliated Hospital of Sun Yat-sen University, and other participating centers have submitted their review results (Approval No.: 2021ZSLYEC-206). We confirm that we have received ethical approval to conduct this study as well as permission for the dataset and that the study was conducted in accordance with the provisions of the Declaration of Helsinki (as amended in Fortaleza, Brazil, October 2013). The data obtained are only collected and analyzed, but the details are not publicly released, and information confidentiality rules are strictly observed.

### Metagenomic sequencing and processing

In this study, genomic DNA was extracted from human fecal samples using the PowerSoil Pro Kit (Qiagen, Germany). DNA libraries were prepared using the NEBNext^®^ Ultra DNA Kit and sequenced on the Illumina NovaSeq platform. After sequencing, low-quality reads and host sequences were removed using fastp [26] and Bowtie2 [27], resulting in 1.61 Tb of high-quality data. The average number of clean reads per sample is approximately 21.8 million. After quality control using KneadData, species identification was performed using Kraken2 (v2.0.7) with the standard RefSeq database [28] and abundance estimation was conducted using Bracken [29]. MetaPhlAn (v4.0.3) [30] and HUMAnN (v3.6) [31] were used for microbial community and metabolic pathway analysis, excluding pathways or species present in fewer than 10 samples. LEfSe [32] analysis was employed to identify significantly different microbial species (P values and adjusted P values < 0.05), with effect size assessed via Linear discriminant analysis (LDA) (LDA score log10 > 2) (Supplementary Table 1). Finally, human DNA content (HDCs) was estimated using Bowtie2 (Supplementary Table 2).

### Construction of the random forest classifier model

For all samples, classification models were developed using the random forest algorithm from the sklearn [33] package in Python 3.6.5. Random forest models were developed using a stratified 80/20 training-testing split. Hyperparameters were optimized using grid search to prevent overfitting. Model performance was rigorously assessed using 100 bootstrap replications for internal validation, and evaluated by AUC, accuracy, sensitivity, specificity, and F1-score. Three classification models were built: (1) high-altitude population vs. coastal population, (2) high-altitude adenoma population vs. healthy high-altitude population, and (3) high-altitude adenoma population vs. coastal adenoma population. In brief, the models were developed using the sklearn package, and the accuracy of the trained classifiers was evaluated using 100 bootstrap resampling. Subsequently, confusion matrices were applied to calculate the evaluation metrics for classification (F1 score, sensitivity, and specificity), and the area under the curve (AUC) was assessed using the pROC package. Additionally, the top 10 most important features, based on the average importance in the random forest model, were extracted and visualized using the ggplot2 [34] package.

### Analysis of MetaCyc metabolic pathways

Using the HUMAnN (v3.6) software, sequences that passed quality control and host removal were aligned with the protein database (UniProt UniRef90) using DIAMOND [35], and reads that failed to align were filtered out. The relative abundance of each protein in UniRef90 was calculated, and relative abundance was also computed using the HUMAnN provided script humann_renorm_table, based on the raw abundance. Since there is a many-to-many relationship between functions and sequences (one sequence can correspond to multiple functions, and one function can correspond to multiple sequences), it is not possible to directly calculate the TPM abundance of functions (as sequence length cannot be corrected). However, UniRef90 and sequences are in a one-to-one relationship, with UniRef90 serving as a broad representation of genes. Using the mapping between UniRef90 IDs and Metacyc [36] database IDs, the abundance of genes (UniRef90) corresponding to the same function was aggregated to obtain the relative abundance of each functional category in the functional database.

### Identification of enterotypes

In this study, a multi-step analysis was conducted using various R packages for each analysis step to explore the classification of gut microbiota enterotypes and their driving factors. First, relative abundance data at the genus level was preprocessed by removing genera with relative abundances below 0.01% to reduce the influence of rare genera. Next, sample similarity was calculated based on Bray-Curtis distance (using the vegan package) [37], and samples were clustered into enterotypes using Partitioning Around Medoids (PAM) (Supplementary Table 3). The optimal number of clusters was determined using the Calinski-Harabasz index and silhouette coefficient. To confirm the clustering and identify the driving factors of enterotype classification units, a Between-Class Analysis (BCA) was performed using the R ade4 package [38] (v.1.7–22). BCA is a special type of principal component analysis (PCA) with instrumental variables, which identifies principal components based on the centroid of each group, highlighting the differences between groups, and links each sample to its respective group. The driving genera for each enterotype were determined as the genera with the highest relative abundance within each enterotype. Subsequently, the PAM clustering method was used to classify fecal samples into fungal and archaea enterotypes, as previously described for bacterial enterotype classification [21, 39]. To validate the robustness of the classification, it was compared to the Dirichlet Multinomial Mixture Model and Jensen-Shannon Divergence distance. Additionally, principal coordinates analysis (PCoA) was performed to visualize the differences between samples and assess the effectiveness of enterotype classification. Finally, the genera with the highest relative abundance in each enterotype were identified and defined as the driving factors of that enterotype.

### Statistical analysis

Statistical analysis was performed using SPSS 27.0. The Mann-Whitney U test was used for comparing differences between two groups, and the Kruskal-Wallis H test was used for comparisons between multiple groups. Pairwise comparisons of cross-boundary microbial abundance were conducted using the Wilcoxon signed-rank test. Taxonomic differences between groups were assessed using PERMANOVA on Bray-Curtis distances via the “adonis” function in the R package “vegan” [40]. Spearman correlation was used to assess the relationships between cross-boundary species communities and MetaCyc metabolic pathways. For comparisons of enterotype characteristics (such as diversity) and host phenotypes (such as age and gender), Fisher’s exact test or the chi-squared test was used for categorical variables, and the Wilcoxon rank-sum test was applied for continuous variables. The Wilcoxon rank-sum test was also used to determine enriched pathways within each enterotype. False discovery rate (FDR) correction was applied to adjust p-values for multiple hypothesis testing in all differential abundance and pathway enrichment analyses, with FDR-adjusted *p*-values < 0.05 considered statistically significant. LEfSe analysis was conducted to identify taxa with significant differences between groups. This involved a non-parametric Kruskal–Wallis test (α = 0.05) followed by linear discriminant analysis (LDA) to estimate effect size. Only features with LDA score > 2.0 and FDR-adjusted p-values < 0.05 were retained. All results were visualized using box plots and bar plots generated with the MATLAB package in Python 3.6.5 [41].

## Results

### Overall characteristics of the enterotype microbial communities of multi-kingdom microbes in coastal and high-altitude populations

**Fig. 1:**
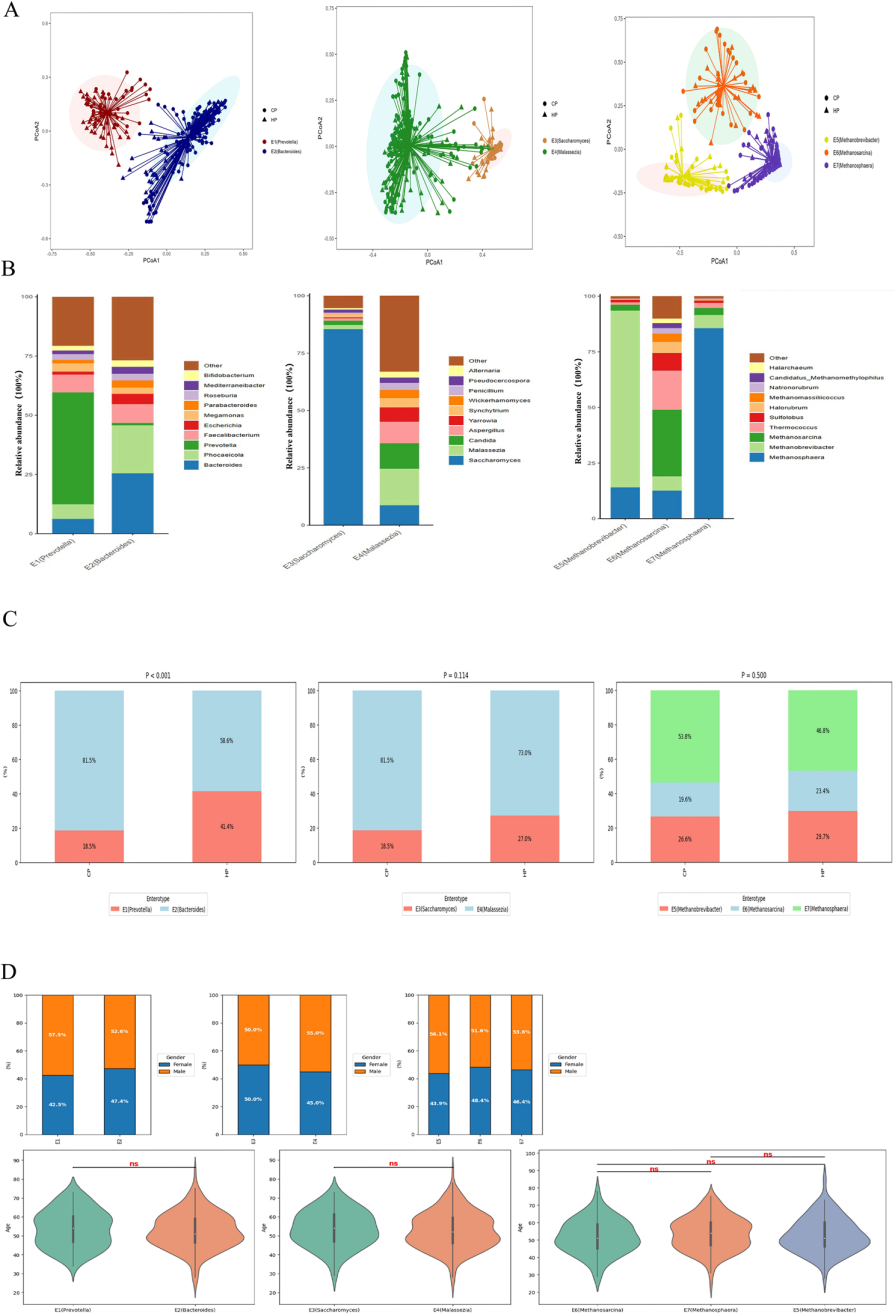
Enterotype analysis of 295 samples of multi-kingdom microorganisms. **A**: The results of enterotype analysis were visualized using PCoA based on the Bray-Curtis distance of genus composition. **B**: Proportion of microbial genera in each enterotype. **C**: the proportion of enterotypes in the two groups of CP and HP. **D**: Distribution of clinical factors such as gender and age in enterotype. ns indicated no significant difference between the two groups (*P* > 0.05)

First, the CP and HP were analyzed using a PCoA plot (Fig. 1A). Then, using JSD distance metrics, clustering of 295 samples based on the relative abundance of bacteria, fungi, and archaea at the genus level was performed. The optimal clustering was determined by considering profile width, CH index, DBI index, and Dunn index, with K being 2, 2, and 6, respectively (Supplementary Fig. 1A). Based on the dominant genera in each group, the two bacterial enterotypes were assigned to Prevotella (E1, *n* = 80) and Bacteroides (E2, *n* = 215). The two fungal enterotypes were assigned to Saccharomyces (E3, *n* = 64) and Malassezia (E4, *n* = 231). The three archaeal enterotypes were assigned to Methanobrevibacter (E5, *n* = 82), Methanosarcina (E6, *n* = 62), and Methanosphaera (E7, *n* = 151). The average read count for fungi accounts for 0.01% of the total reads per sample, and for archaea, it is 0.08%, reflecting their relatively low abundances compared to bacteria (97%) in the shotgun metagenomic data. The proportions of cross-boundary microbial genera within each enterotype are shown in Fig. 1B. In brief, *Prevotella* (46.98%) and *Faecalibacterium* (7.72%) were relatively abundant in the E1 enterotype. *Bacteroides* (25.26%) and *Phocaeicola* (20.12%) were relatively abundant in the E2 enterotype. *Saccharomyces* (85.45%) was relatively abundant in the E3 enterotype. *Malassezia* (15.95%) and *Candida* (11.10%) were relatively abundant in the E4 enterotype. Methanobrevibacter (80.41%) was relatively abundant in the E5 enterotype. *Methanosarcina* (30.82%) and *Thermococcus* (17.45%) were relatively abundant in the E6 enterotype. *Methanosphaera* (86.85%) dominated the E7 enterotype (Fig. 1B). Further analysis of the proportions of cross-boundary microbial enterotypes in the coastal and high-altitude populations showed that in the high-altitude population, the bacterial enterotype E1 was more prevalent than in the coastal population (41.4% vs. 18.5%), while the fungal enterotype E3 (27.0% vs. 18.5%) and the archaeal enterotype E5 (29.7% vs. 26.6%) also showed higher proportions in the high-altitude population (Fig. 1C). Additionally, we analyzed the distribution of clinical factors such as age and sex across the cross-boundary microbial enterotypes. The results showed no statistically significant differences in these clinical factors across the enterotypes (*P* > 0.05) (Fig. 1D). Furthermore, analysis of the grouped populations in relation to age and sex revealed no significant differences (Supplementary Table 4).

### Genus-level (bacterial) composition of the E1 enterotype gut microbiomes across the two regions

**Fig. 2:**
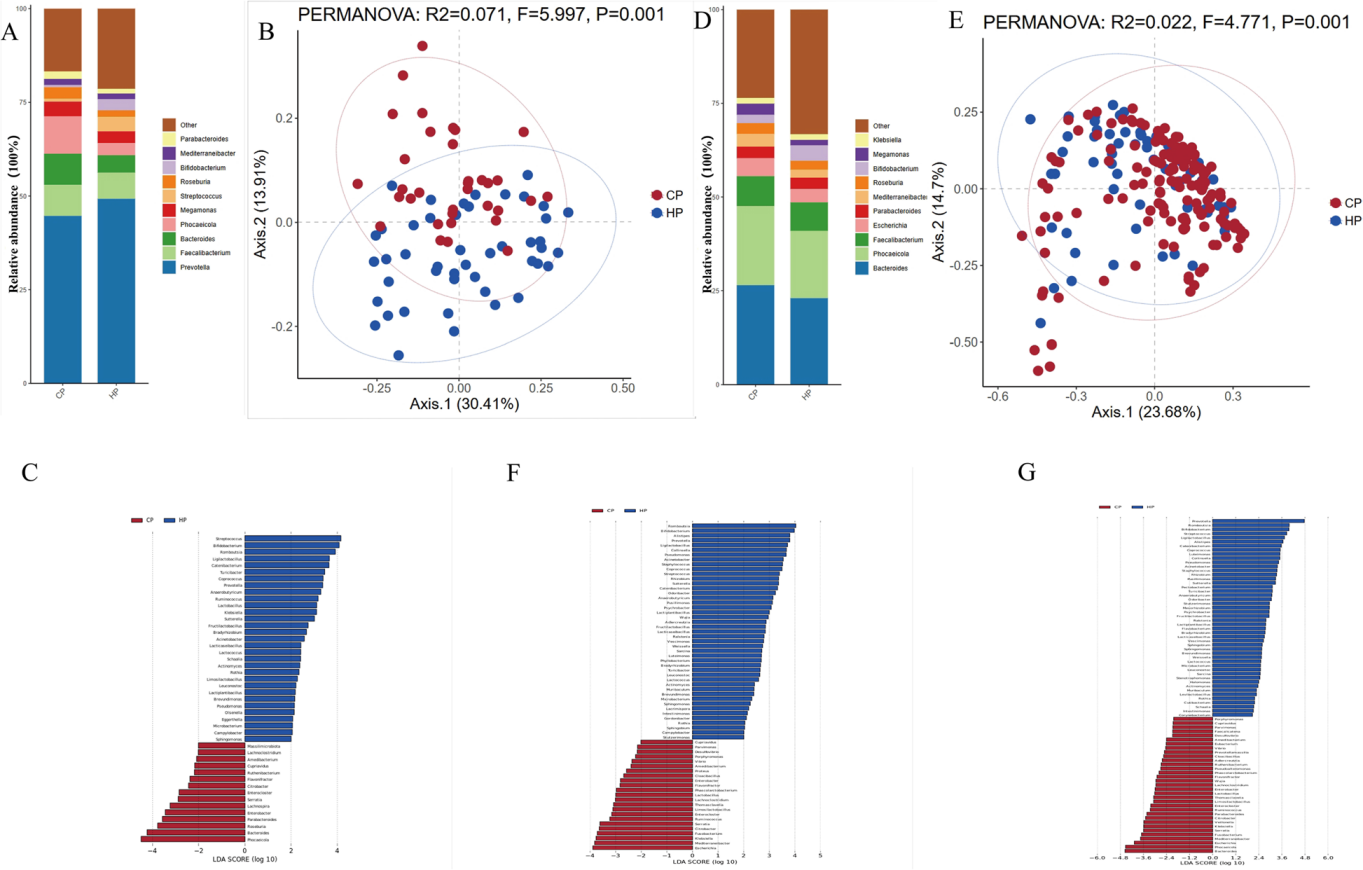
Different bacterial compositions of E1 and E2 enterotype samples from CP and HP. **A**: Abundance of gut microbiota communities at the genus level for different bacterial compositions of E1 enterotype samples. **B**: Principal component analysis (PCA) plot of the E1 enterotypes sample visualizing two human cohorts. Red and blue dots represent CP and HP, respectively. **C**: E1 enterotype samples were found to have differentially abundant bacterial genera in CP and HP samples by LDA. **D**: Abundance of gut microbiota communities at the genus level for the different bacterial composition of the E2 enterotype samples. **E**: PCA plot of the E2 enterotype sample visualizing two human cohorts. **F**: E2 enterotype samples were found to have differentially abundant bacterial genera in CP and HP samples by LDA. **G**: Differences in bacterial gut microbiota characteristics between CP and HP based on all samples

To investigate the gut microbiome composition and its functional associations across different populations, we first stratified the samples by enterotype. Enterotypes represent distinct gut microbial community structures, which may exhibit different taxonomic and functional patterns. In the E1 enterotype, the genus-level bacterial composition of the two regional populations is shown in Fig. 2A. In both populations, *Prevotella*,* Faecalibacterium*,* Bacteroides*,* Phocaeicola*, and *Megamonas* were identified as the five dominant genera. The PCA plot shows a significant difference in the bacterial composition between the two populations (*P* < 0.005) (Fig. 2B). Further analysis using LDA to screen for differences in microbial species between the two populations revealed 46 genera with significant differences between the CP and HP groups (Fig. 2C). Among them, 15 genera, including *Phocaeicola*,* Bacteroides*, and *Roseburia*, were significantly enriched in the CP group; while 31 genera, including *Streptococcus*,* Bifidobacterium*, and *Romboutsia*, were significantly enriched in the HP group.

### Genus-level (bacterial) composition of the E2 enterotype gut microbiomes across the two regions

In the E2 enterotype, Fig. 2D shows the relative abundance of bacterial genera at the genus level for the two regional populations. In both populations, *Bacteroides*,* Phocaeicola*,* Faecalibacterium*,* Escherichia*, and *Parabacteroides* are the five major bacterial genera. The PCA plot shows a significant separation in the bacterial composition between the two groups (*P* < 0.005) (Fig. 2E). Additionally, the LDA method was used to screen for specific genera in each group (Fig. 2F). In short, 23 genera were significantly enriched in the CP group, including *Escherichia*,* Mediterraneibacter*, and *Klebsiella;* while 45 genera were primarily identified in the HP group, such as *Romboutsia*,* Bifidobacterium*,* and Alistipes*. To verify the reliability of the enterotype classification criteria, we also analyzed all samples from different regional populations without performing enterotype classification. The results showed significant differences in 81 intestinal genera between the two groups (Fig. 2G). In brief, 48 bacterial genera, including *Prevotella*,* Romboutsia*,* and Bifidobacterium*, were significantly enriched in the HP group, while 33 microbial markers, including *Bacteroides*,* Phocaeicola*,* and Escherichia*, were most abundant in the CP group.

### Genus-level (fungal) composition of the E3 enterotype gut microbiomes across the two regions

**Fig. 3:**
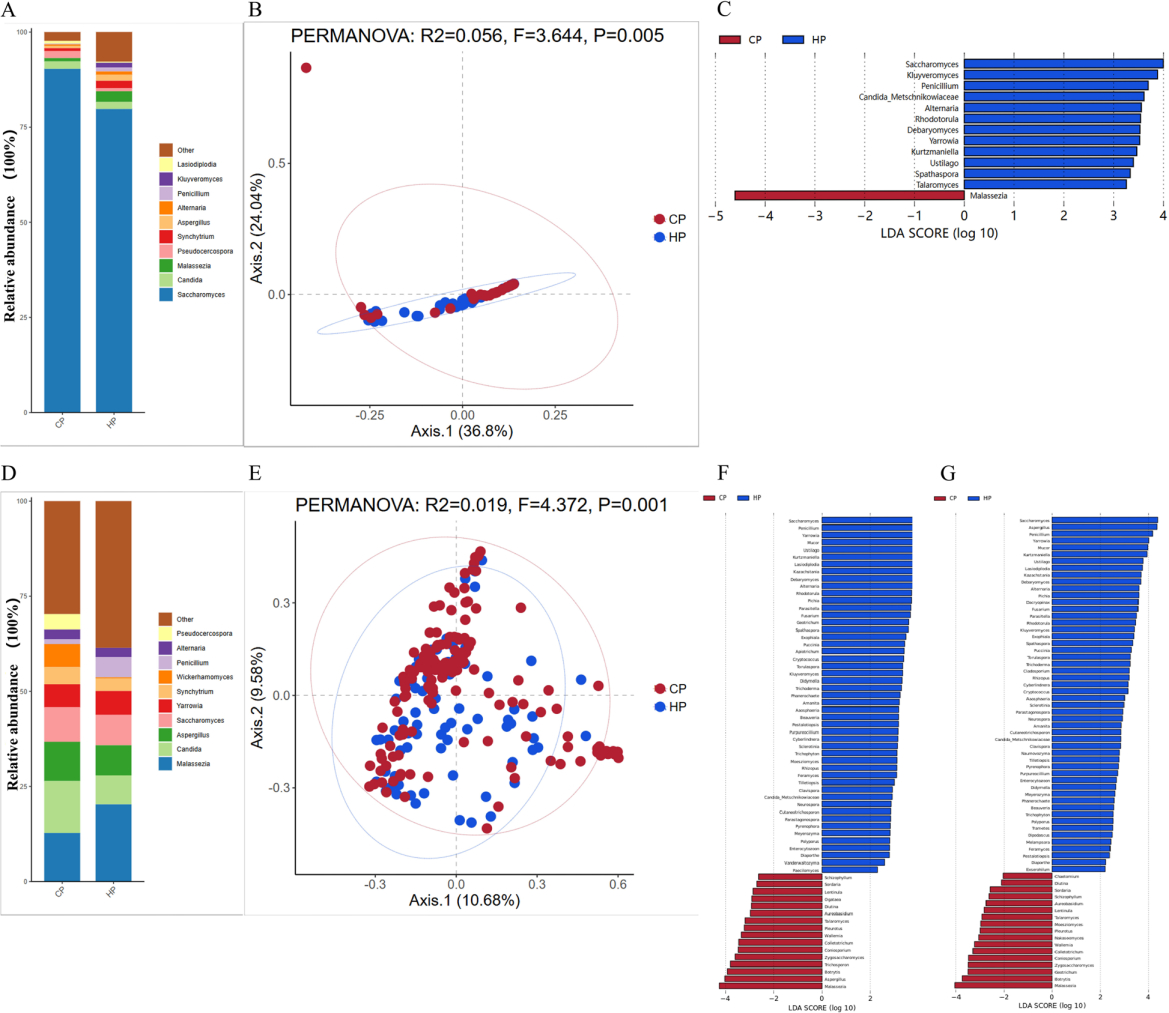
Different fungal composition of E3 and E4 enterotype samples from CP and HP. **A**: Gut microbiota community abundance at the genus level for different fungal compositions of E3 enterotype samples. **B**: PCA plot of the E3 enterotype sample visualizing two human cohorts. Red and blue dots represent CP and HP, respectively. **C**: E3 enterotype samples were found to have differentially abundant fungal genera in CP and HP samples by LDA. **D**: Abundance of gut microbiota communities at the genus level for different fungal compositions in the E4 enterotype sample. **E**: PCA plot of the E4 enterotype sample visualizing two human cohorts. **F**: E4 enterotype samples were found to have differentially abundant fungal genera in CP and HP samples by LDA. **G**: Differences in fungal gut microbiota characteristics between CP and HP based on all samples

In the E3 enterotype, Fig. 3A shows the fungal composition at the genus level for the two regional populations. In both populations, *Saccharomyces*,* Candida*,* Malassezia*,* Pseudocercospora*, and *Synchytrium* were identified as the five dominant genera. The PCA plot shows a significant difference in the community composition between the two groups (*P* < 0.005) (Fig. 3B). Further analysis using LDA to identify species differences in the microbiota between the two groups found 13 genera with differences between CP and HP (Fig. 3C). Among these, 12 genera, including *Saccharomyces*,* Kluyveromyces*, and *Penicillium*, were significantly enriched in the HP group. In brief, only one genus, *Malassezia*, was significantly enriched in the CP group.

### Genus-level (fungal) composition of the E4 enterotype gut microbiomes across the two regions

In the E4 enterotype, Fig. 3D shows the relative abundance of fungal genera at the genus level for the two regional populations. In both populations, *Malassezia*,* Candida*,* Aspergillus*,* Saccharomyces*, and *Yarrowia* were identified as the five dominant genera. The PCA plot shows a significant separation in community composition between the two groups (*P* = 0.001) (Fig. 3E). Further analysis using LDA to identify specific genera for each group found 16 genera significantly enriched in the CP group, including *Malassezia*,* Aspergillus*, and *Botrytis.* In the HP group, 49 genera were primarily identified, such as *Saccharomyces*,* Penicillium*, and *Yarrowia* (Fig. 3F). To verify the reliability of the two enterotype classification standards, we also analyzed all the samples from different regional populations without the classification into enterotypes. The results showed that the two groups of subjects had differences in 81 genera of intestinal microbiota (Fig. 3G). In brief, 53 fungal genera, including *Saccharomyces*,* Aspergillus* and *Penicillium*, were significantly enriched in the HP group, while 15 microbial markers, including *Malassezia*,* Botrytis*, and *Geotrichum*, were most abundant in the CP group.

### Genus-level (archaeal) composition of the E5 enterotype gut microbiomes across the two regions

**Fig. 4:**
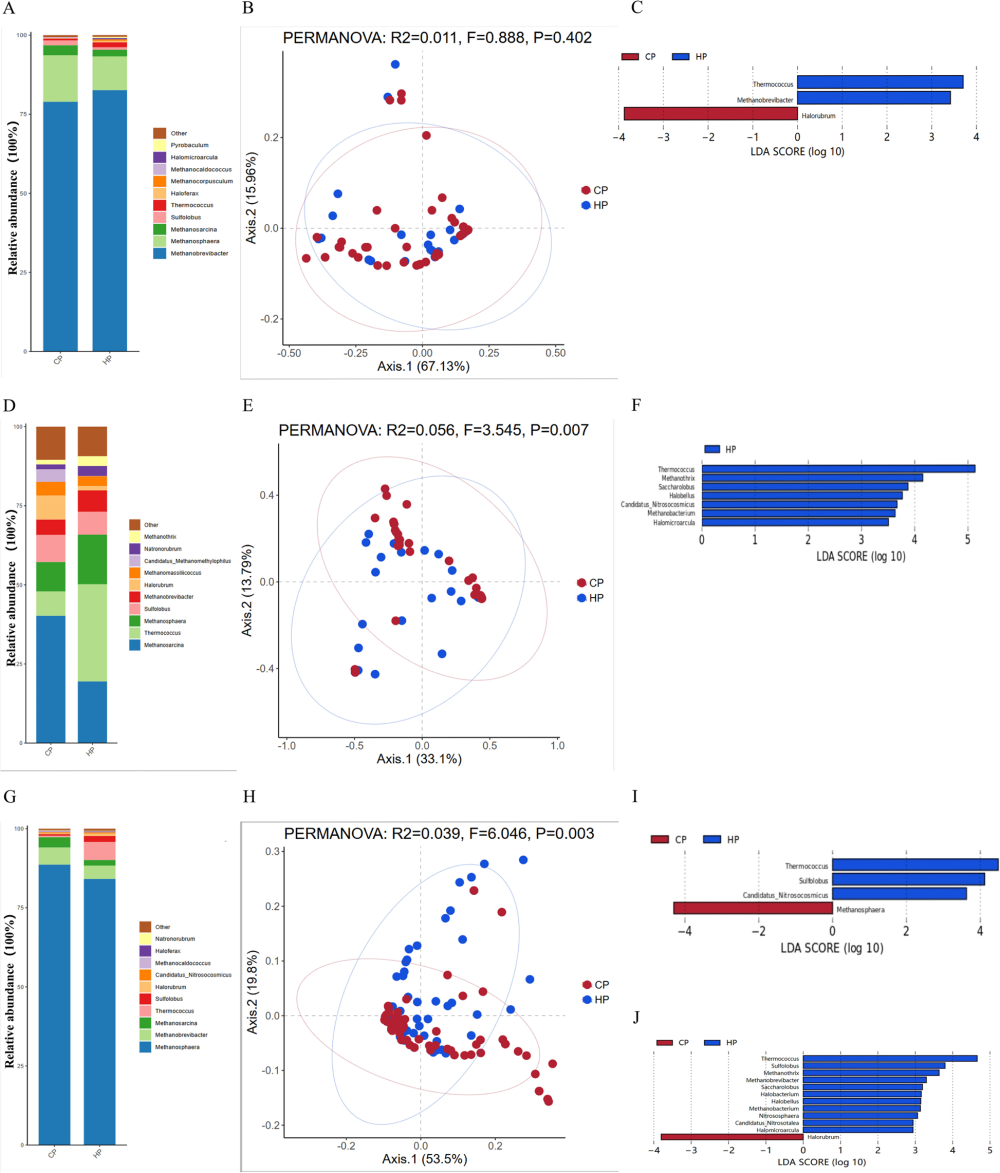
Different archaeal composition of E5, E6, and E7 enterotype samples from CP and HP. **A**: Abundance of gut microbiota communities at the genus level for different archaeal compositions of E5 enterotype samples. **B**: PCA plot of E5 enterotype samples visualizing two human cohorts. Red and blue dots represent CP and HP, respectively. **C**: E5 enterotype samples were found to have differentially abundant archaeal genera in CP and HP samples by LDA. **D**: Abundance of gut microbiota communities at the genus level for the different archaeal composition of E6 enterotype samples. **E**: PCA plot of the E6 enterotype sample visualizing two human cohorts. **F**: E6 enterotype samples were found to have differentially abundant archaeal genera in CP and HP samples by LDA. **G**: Abundance of gut microbiota communities at the genus level for the different archaeal composition of E7 enterotype samples. **H**: PCA plot of E7 enterotype samples visualizing two human cohorts. **I**: E7 enterotype samples identified differentially abundant archaeal genera in CP and HP samples by LDA. **J**: Differences in archaeal gut microbiota characteristics between CP and HP based on all samples

In the E5 enterotype, Fig. 4A shows the genus-level archaeal composition for the two regional populations. In both populations, *Methanobrevibacter*,* Methanosphaera*,* Methanosarcina*,* Sulfolobus*, and *Thermococcus* were identified as the five dominant genera. The PCA plot shows no significant difference in community composition between the two groups (*P* = 0.402) (Fig. 4B). Further analysis using LDA to identify specific genera for each group found 3 genera with differences between the CP and HP groups (Fig. 4C). Among them, *Thermococcus and Methanobrevibacter* were significantly enriched in the HP group; in contrast, only one genus, *Halorubrum*, was significantly enriched in the CP group.

### Genus-level (archaeal) composition of the E6 enterotype gut microbiomes across the two regions

In the E6 enterotype, Fig. 4D shows the genus-level archaeal composition for the two regional populations. In both populations, *Methanosarcina*,* Thermococcus*,* Methanosphaera*,* Sulfolobus*, and *Methanobrevibacter* were identified as the five major archaeal genera. The PCA plot shows a significant separation between the two groups (*P* = 0.007) (Fig. 4E). Further analysis using LDA to identify specific genera for each group (Fig. 4F) found 7 genera significantly enriched in the HP group, including *Thermococcus*,* Methanothrix*, and *Saccharolobus.*

### Genus-level (archaeal) composition of the E7 enterotype gut microbiomes across the two regions

In the E7 enterotype, Fig. 4G shows the genus-level archaeal composition for the two regional populations. In both populations, *Methanosphaera*,* Methanobrevibacter*,* Methanosarcina*,* Thermococcus*, and *Sulfolobus* were identified as the five major archaeal genera. The PCA plot shows a significant separation between the two groups (*P* = 0.003) (Fig. 4H). Further analysis using LDA to identify specific genera for each group (Fig. 4I) found 3 genera significantly enriched in the HP group, including *Thermococcus*,* Sulfolobus*, and *Candidatus_Nitrosocosmicus.* Only one genus, *Methanosphaera*, was significantly enriched in the CP group. To validate the reliability of the three enterotype classification standards, we also analyzed all samples from different regional populations without the enterotype classification. The results showed that the two groups differed in 12 gut bacterial genera (Fig. 4J). Specifically, 11 genera (including *Thermococcus*,* Sulfolobus*, and *Methanothrix*) were significantly enriched in the HP group, and 1 microbial marker (*Halorubrum*) had the highest abundance in the CP group.

### Functional differences of enterotypes across groups within each microbial kingdom

**Fig. 5:**
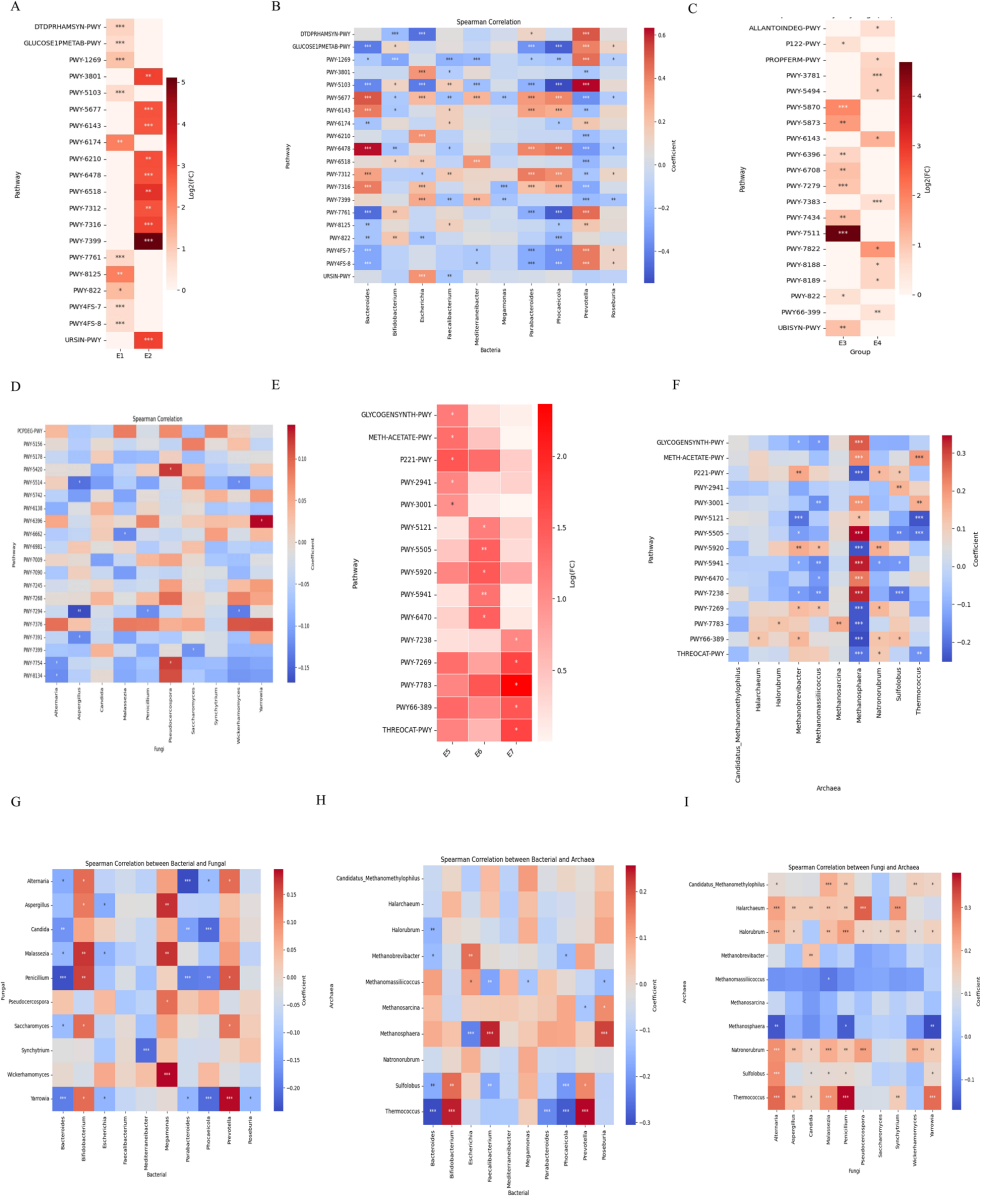
Metabolic pathways associated with enterotypes of multi-kingdom microbes. **A**-**B**: Bacterial pathways enriched in different bacterial enterotypes (**A**) and related bacterial genera (**B**). **C**-**D**: Bacterial pathways enriched in different fungal enterotypes (**C**) and related fungal genera (**D**). **E**-**F**: Bacterial pathways enriched in different archaeal enterotypes (**E**) and associated individual bacterial genera (**F**). **G**: Correlation between fungal and bacterial enterotypes in CP and HP. **H**: Correlation between archaeal and bacterial enterotypes in CP and HP. **I**: Correlation between archaeal and fungal enterotypes in CP and HP. Log(FC) represents the log-transformed fold change of the mean relative abundance of the pathway relative to the other pathways in each multi-kingdom microbial enterotype. Asterisks indicate the statistical significance of the multiple testing corrected Pearson correlation test (top) and the multiple testing corrected Wilcoxon rank-sum test (bottom) : * adjusted *P* < 0.05, ** adjusted *P* < 0.01, and *** adjusted *P* < 0.001

To characterize the bioactivity potential of bacterial enterotypes and their potential metabolic mechanisms, we utilized the MetaCyc gene function annotation database to predict the metabolic functions of gut microbiota in both groups based on the relative abundance of bacteria, fungi, and archaea at the genus level. A total of 20 bacterial, 20 fungal, and 20 archaeal pathways were identified, with different distributions across the enterotypes (using Wilcoxon rank-sum test with adjusted *P* < 0.05; Fig. 5A; Tables S2, S4, and S6 in Supplementary Table 5). Additionally, the relative abundance of these pathways was significantly correlated with the relative abundance of the corresponding 10 genera (*P* < 0.05, Pearson correlation; Fig. 5B; Tables S3, S5, and S7 in Supplementary Table 5). Consistent with previous studies, the E1 enterotype showed enrichment of carbohydrate degradation pathways (GLUCOSE1PMETAB-PWY, *P* < 0.05), reflecting adaptation to fiber-rich, plant-based diets common in high-altitude populations (Fig. 5A). Consistent with previous findings that Bacteroides and Phocaeicola dominate the Bacteroides enterotype, our results showed that these genera, along with Prevotella, were significantly associated with the GLUCOSE1PMETAB-PWY pathway, supporting their role in carbohydrate metabolism (Fig. 5B). In the E3 enterotype, the generation of 2,3-butanediol, a key metabolic pathway for many gut microbes under hypoxic or anaerobic conditions, was observed. This pathway helps reduce the accumulation of acidic metabolites and maintain the balance of the gut microenvironment. Considering that systemic hypoxia associated with high altitudes can cause changes in the host’s metabolism and immune function, this microbial metabolic function may indirectly promote the homeostasis of the enterotypes and help the host adapt to the plateau environment. Overexpression of the 2,3-butanediol biosynthesis pathway (PWY-6396, *P* < 0.05) was noted (Fig. 5C). Furthermore, Saccharomyces, the dominant genus in the E3 enterotype, was significantly correlated with the degradation of methylphosphonic acid (Fig. 5D). In the E5 enterotype, pathways related to isoleucine biosynthesis (PWY-3001, *P* < 0.05) were overexpressed, indicating a potential enhancement of amino acid metabolism in HP. Given isoleucine’s known role in energy and immune regulation, this may reflect an adaptive microbial function in response to high-altitude conditions (Fig. 5E). Additionally, Methanobrevibacter, the dominant genus in the E5 enterotype, was significantly correlated with the heme biosynthesis pathway (PWY-5920, Fig. 5F). This could be linked to changes in gut microbiota composition in hypoxic environments, where hypoxia may alter the metabolic patterns of the microbiota, influencing energy acquisition and metabolic products differently from other populations. Additionally, to explore the relationship between fungal and bacterial enterotypes, we conducted a correlation analysis using the top 10 genera from bacterial and fungal enterotypes. We found a significant correlation between fungal and bacterial enterotypes (*P* < 0.05, Fig. 5G). The dominant genus Prevotella in the bacterial E1 enterotype was positively correlated with the dominant genus Saccharomyces in the fungal E3 enterotype, providing evidence of significant interactions between fungal and bacterial communities. Further, to explore the relationship between archaea, bacteria, and fungal enterotypes, we performed a correlation analysis using the top 10 genera from archaea, bacteria, and fungal enterotypes. A significant correlation was observed between archaea and bacterial enterotypes (*P* < 0.05, Fig. 5H), with the dominant genus Prevotella in the bacterial E1 enterotype positively correlated with Thermococcus, the dominant genus in the archaeal E5 enterotype. Additionally, our results showed a significant correlation between archaea and fungal enterotypes (*P* < 0.05, Fig. 5I), with the dominant genus Malassezia in the fungal E3 enterotype positively correlated with Thermococcus, the dominant genus in the archaeal E5 enterotype. These findings suggest significant correlations between archaea, fungi, and bacterial communities.

### Enterotypes for distinguishing populations from different regions using random forest classification

**Fig. 6:**
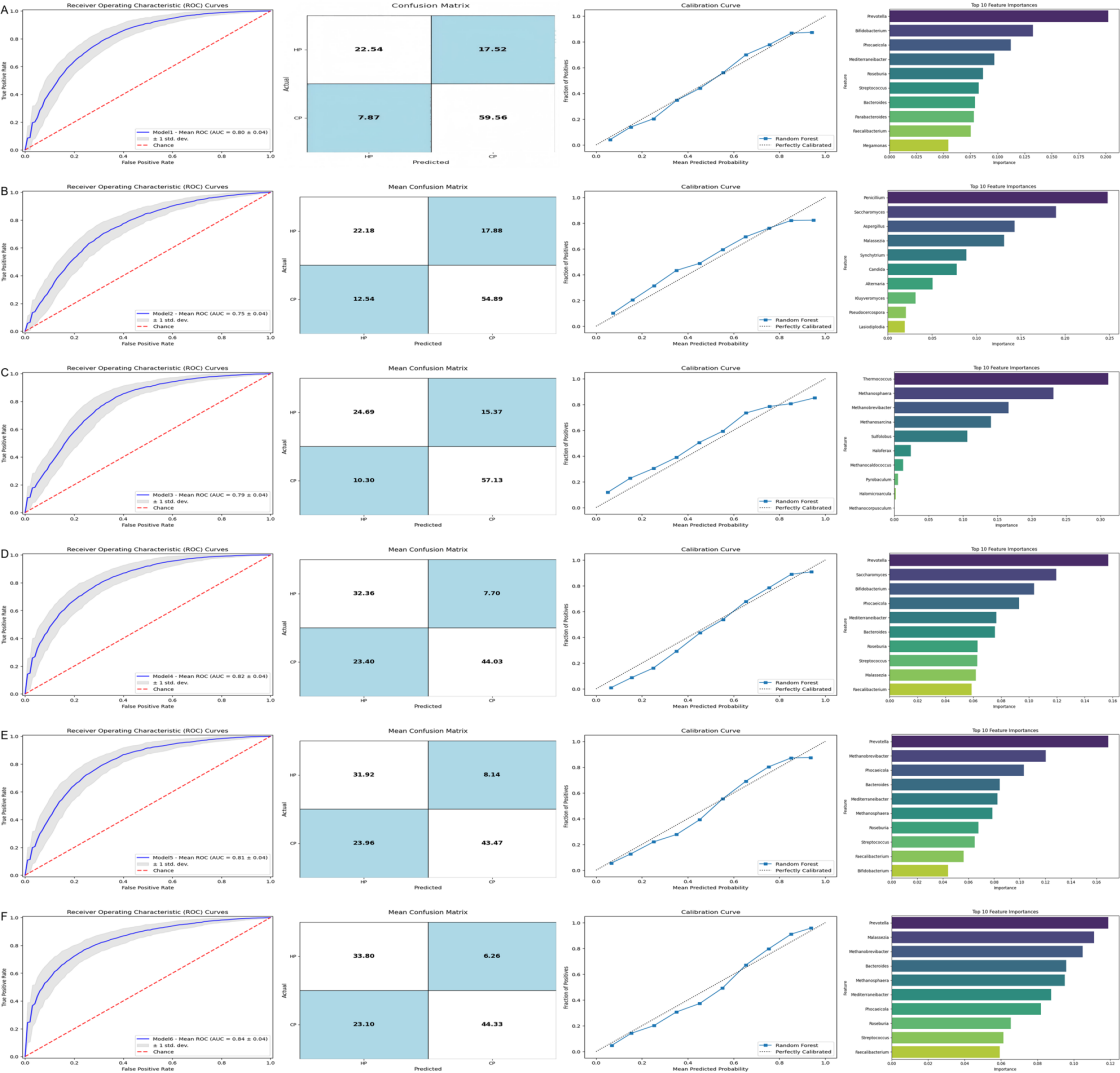
**A**: Prediction model based on the 10 bacteria enriched in CP and HP. The average ROC curve shows the performance of the prediction model, the average confusion matrix shows the classification performance of the model, the calibration curve shows the agreement between the predicted probability and the actual results, and the bar graph shows the average feature importance of the 10 bacterial species used in the prediction model. **B**: Prediction model based on 10 fungi enriched in CP and HP. **C**: Prediction model based on 10 archaeal species enriched in CP and HP. **D**: Diagnostic performance of a model combining bacterial and fungal microbial markers to distinguish CP from HP. **E**: Diagnostic performance of models incorporating bacterial and archaeal microbial markers to distinguish CP from HP. **F**: Diagnostic performance of a model incorporating bacterial, fungal, and archaeal microbial markers to distinguish CP from HP

Given that we observed differences in the composition of enterotypes among the subject groups, we further investigated whether these enterotypes could be used to differentiate populations from the high-altitude and coastal regions. We evaluated the predictive ability of binary classification models for identifying high-altitude and coastal populations. Our results showed that the dominant bacterial enterotype in the high-altitude population is E1, so we used the top 10 dominant bacterial genera from E1 to construct a random forest model for distinguishing HP. The classification AUC was 0.80 (F1 score = 0.824), with sensitivity and specificity of 0.773 and 0.741, respectively. Additionally, we found that the feature Prevotella had the highest importance score (Fig. 6A). Similarly, the dominant fungal enterotype in the high-altitude population is E3, and we constructed a random forest model using the top 10 dominant fungal genera from E3 for distinguishing HP. The classification AUC was 0.75 (F1 score = 0.78), with sensitivity and specificity of 0.81 and 0.56, respectively (Fig. 6B). For the archaeal enterotype, the dominant enterotype in the high-altitude population is E5, so we used the top 10 dominant archaeal genera from E5 to build a random forest model for distinguishing HP. The classification AUC was 0.79 (F1 score = 0.82), with sensitivity and specificity of 0.85 and 0.61, respectively (Fig. 6C). To assess whether a multi-kingdom microbiome-based model would perform better, we combined the top 10 dominant bacterial genera from E1 and the top 10 dominant fungal genera from E3, which resulted in an AUC value of 0.82, surpassing the performance of the bacterial-only model (AUC = 0.80; Fig. 6D). Similarly, combining the top 10 dominant bacterial genera from E1 and the top 10 dominant archaeal genera from E5 resulted in an AUC value of 0.81, outperforming the archaeal-only model (AUC = 0.79; Fig. 6E). Furthermore, we evaluated whether a multi-kingdom microbiome-based model, incorporating bacterial, fungal, and archaeal enterotypes, would provide better predictive value. By combining the top 10 dominant bacterial genera from E1, the top 10 dominant fungal genera from E3, and the top 10 dominant archaeal genera from E5, the AUC value increased to 0.84 (Fig. 6F), which outperformed models using only bacteria, fungi, or archaea, as well as combinations of two enterotype types. In conclusion, these results highlight the enhanced classification predictive ability for distinguishing high-altitude and coastal populations by integrating bacterial, fungal, and archaeal microbial markers.

### Subgroup analysis of intestinal microflora in coastal adenoma population and high-altitude adenoma population

To further investigate the relationship between populations from two regions and CRA, our study conducted a subgroup analysis comparing the CAP and the HAP. Our results revealed that, compared to the CAP group, the cross-kingdom microbiota enterotypes in the HAP group were clustered as follows: Prevotella (E1, *n* = 37) and Bacteroides (E2, *n* = 113), Saccharomyces (E3, *n* = 29) and Malassezia (E4, *n* = 121), as well as Methanobrevibacter (E5, *n* = 29), Methanosarcina (E6, *n* = 33), and Methanosphaera (E7, *n* = 88) (Supplementary Fig. 2A). The relative abundance of cross-kingdom microbial genera in each enterotype is shown in Supplementary Fig. 2B. Our findings also indicated that in the HAP group, the bacterial enterotype E1 was more prevalent than in the CAP group (36.4% vs. 19.8%), the fungal enterotype E3 was more prevalent (27.3% vs. 16.0%), and the archaeal enterotype E5 was more prevalent (25.0% vs. 17.0%) (Supplementary Fig. 2C). We used the cross-kingdom microbiota enterotypes to distinguish between CAP and HAP by constructing a random forest model. We integrated the top 10 dominant genera from bacterial enterotype E1, fungal enterotype E3 and archaeal enterotype E5 to build a multi-domain classification model. This model achieved an AUC of 0.85, outperforming single-kingdom models which had AUC values of 0.79 for bacteria, 0.64 for fungi and 0.68 for archaea, indicating good classification performance. These key results about CAP and HAP distinction via cross-kingdom enterotype models are detailed in Supplementary Text 1. Furthermore, to explore the impact of the high-altitude environment on CRA occurrence, we performed a second subgroup analysis comparing the High-altitude Control Population (HCP) and the HAP. Key findings from this subgroup analysis include distinct enterotype distributions between HCP and HAP, significant differences in genus-level microbial composition (such as *Malassezia* and *Thermococcus* enriched in HAP, *Roseburia* and *Blautia* enriched in HCP) and functional metabolic pathway adaptations related to hypoxia resistance and energy metabolism. The detailed results of this HCP vs. HAP analysis are provided in Supplementary Text 2.

## Discussion

Due to the significant inter-individual variability in gut microbiota composition, the concept of enterotypes is crucial in microbiome-related research. Enterotypes help stratify human populations based on gut microbiota composition, with each enterotype having a distinct bacterial makeup, making it a reliable method for understanding the gut microbiota community that is not influenced by factors like age, sex, or ethnicity. Currently, enterotypes have mainly been described in healthy individuals or patients without cancer, such as the “enterotype” concept proposed by Frioux et al. [42], which is characterized by specific microbial taxa. However, studies investigating enterotype variations from regional and disease status perspectives are limited. In this study, we classified the gut microbiota of participants into several enterotypes based on the similarity of bacterial, fungal, and archaeal compositions. First, the cohort was divided into high-altitude and coastal populations. In the bacterial enterotypes, two types emerged (E1: Prevotella enterotype and E2: Bacteroides enterotype). Further analysis revealed significant changes in the composition, ecology, and functionality of bacteria, fungi, and archaea from healthy individuals to adenoma patients in both coastal and high-altitude regions. Based on these findings, we developed a set of microbial markers combining fungal, archaeal, and bacterial features to distinguish between high-altitude and coastal populations, as well as between adenoma patients and healthy individuals. Our study showed that the E1 enterotype, dominated by Prevotella, was more prevalent in the high-altitude population (41.4% vs. 18.5%, *P* < 0.05) (Fig. 1D), which aligns with previous research indicating that the Tibetan population’s gut microbiota is characterized by a relative abundance of Prevotella [15]. Furthermore, based on LDA analysis, Prevotella was identified as a characteristic bacterium of the Prevotella enterotype and a potential microbial marker for both HP and HAP. Prevotella is a Gram-negative bacterium that exhibits higher abundance in HP than in CP and higher abundance in HAP than in CAP. This indicates a possible trend toward an inverse relationship between Prevotella abundance and CRA presence in both HP and HAP, which warrants further dedicated analysis. Species-level analysis (Figure S13) shows significant *Prevotella copri* enrichment in the HP group, consistent with its association with highland diets rich in complex carbohydrates. Further species-level analysis reveals that *Prevotella copri*, a representative species of the genus closely associated with carbohydrate metabolism, may adapt to the traditional high-carbohydrate, low-fat, and low-protein dietary pattern of high-altitude populations through enhancing the ability to ferment dietary fiber into short-chain fatty acids (SCFAs). This dietary pattern is prevalent among non-Westernized populations, including those in high-altitude regions [43]. Multiple studies have shown that Prevotella is one of the core microbiota in Tibetan populations, with its high abundance related to high carbohydrate intake and low-fat and low-protein consumption [19, 21]. The Prevotella enterotype mainly consists of fiber-utilizing bacteria that ferment dietary fibers into SCFAs [44]. A recent cohort study in the U.S. demonstrated that increasing fiber intake after a CRA diagnosis can reduce mortality, suggesting that Prevotella may benefit CRA prognosis. This is because dietary fiber promotes the production of SCFAs, which help maintain intestinal barrier function and reduce inflammation [45]. Additionally, a cohort study in Korea showed that the typical Prevotella enterotype aids in improving CRA prognosis, possibly due to its association with low levels of histamine-degrading enzymes in CRA tissues [46]. Furthermore, Prevotella is crucial for maintaining gut homeostasis in high-altitude regions [15, 18]. Our results further highlight that the Prevotella enterotype has high precision (AUC = 0.80) in distinguishing HP from CP and coastal adenoma patients from high-altitude adenoma patients. Microbial metabolites may play an essential role in regulating host health by participating in host metabolism [47]. The microbiota can utilize indigestible carbohydrates in the colon to produce SCFAs, such as acetate, propionate, and butyrate. Our results indicate that the gut microbiome composition differs between coastal and high-altitude populations. While these differences may be associated with altitude-related factors, the underlying mechanisms remain unclear and warrant further investigation. Similarly, when we further clustered the fungal enterotypes, our results showed two distinct fungal enterotypes (E3: Saccharomyces enterotype and E4: Malassezia enterotype) in both HP vs. CP and HAP vs. CAP groups. Among these, the E3 enterotype, dominated by Saccharomyces, was the predominant enterotype in both HP and HAP. Further analysis using LDA identified Saccharomyces as the characteristic fungal of the Saccharomyces enterotype, a potential microbial marker for both HP and HAP. *Saccharomyces*, a commensal yeast, showed relatively higher abundance in HP compared to CP, and in HAP compared to CAP. Although these findings indicate potential differences in fungal community composition, their clinical significance requires further validation.

Archaea are prokaryotic, single-celled microorganisms, and their collective genetic material in specific environments is referred to as the archaea microbiome [48]. Archaea are primarily considered extremophiles, living in environments vastly different from human habitats and microbial ecosystems. However, with advances in molecular techniques, particularly DNA sequencing and metagenomics, the presence of archaea in non-extreme environments has been revealed [49]. In this study, we clustered the archaea enterotype and observed three primary archaeal clusters dominated by *Methanobrevibacter*,* Methanosarcina*, and *Methanosphaera*. During subgroup analysis, we found that Methanobrevibacter (E5 enterotype) was more abundant in HCP compared to HAP, and it was also more abundant in HAP compared to CAP. At the genus level, we observed an enrichment of several archaeal genera, including *Methanobrevibacter* and *Thermococcus*, in both HP and HAP. Further LDA analysis highlighted *Methanobrevibacter* as a characteristic genus of the Methanobrevibacter enterotype, distinguishing HP and HAP populations. *Methanobrevibacter* is a Gram-negative bacterium whose abundance is higher in HP than in CP, and higher in HAP than in CAP. This suggests that *Methanobrevibacter* may be associated with a lower prevalence of CRA in high-altitude populations. Methanogens contribute significantly to methane production and may be diet-related, influencing disease-related processes [50], with their numbers reduced in conditions such as Crohn’s disease, ulcerative colitis, and severe acute malnutrition [51]. A study on a Chinese population showed that compared to healthy individuals, the abundance of halophilic archaea increased in colorectal cancer, while methanogens decreased [52]. The human colon is anaerobic, and methanogens make up about 10% of the gut’s anaerobic bacteria, playing an active role in enhancing digestive efficiency through methane production [53] and removing harmful metabolites like trimethylamine [54]. Furthermore, the human methane production process primarily relies on hydrogen to reduce carbon dioxide and methyl compounds [55]. Studies have shown that the reduction of hydrogen concentrations by methanogens decreases sulfite levels produced by sulfate-reducing bacteria, thus reducing potential damage to colon epithelial cells [56]. Additionally, it has been reported that African natives with high levels of methanogenic archaea have a lower susceptibility to sporadic colorectal cancer [57]. As our study indicates, the Methanobrevibacter enterotype, dominated by methanogenic archaea, is prevalent in both HP populations and HAP, with species such as *Methanobrevibacter* and *Thermococcus* significantly enriched in comparison to coastal and CAP populations. The consumption of methanogens in the gut may facilitate the development of colorectal cancer, supporting our findings and potentially explaining the lower incidence of CRA in high-altitude regions. Further research has indicated that the enzyme BgaH correlates negatively with CRA. After studying its enzymatic function, it was found to primarily use β-D-galactosidase lactulose as a substrate, thereby inhibiting harmful bacteria and enhancing the production of beneficial metabolites such as SCFAs [58]. Additionally, the enzyme methanogen homoaconitase large subunit is negatively correlated with colorectal adenoma incidence. This enzyme is crucial for the biosynthesis of leucine, an amino acid recently found to be negatively correlated with CRA [59]. In our differential microbiota analysis, *Halorubrum* was significantly enriched in CP and CAP. *Halorubrum*, a genus of halophilic archaea, belongs to the haloarchaea and typically thrives in high-salt environments. Studies have found that compared to healthy individuals, patients with CRA have an increased abundance of halophilic archaea [60]. Halophilic archaea are chemoorganotrophic organisms that grow best in high-salt conditions but can also perform anaerobic respiration in oxygen-deprived environments, and they have been found in human feces and gut mucosa [61]. Environmental factors, such as diet, may play a key role in the mucosal-associated archaea, suggesting that halophilic archaea can colonize the human gut and may contribute to gastrointestinal diseases like CRA. This is potentially linked to the high-salt diet in coastal areas, where the intake of salty food supports the enrichment of halophilic archaea in the gut, which may be related to gut microbiota regulation, obesity, and CRA.

The mutualistic symbiosis between archaea and bacteria has been reported in previous studies [62]. Our study observed that methanogenic archaea *Methanobrevibacter* and *Thermococcus*, which are depleted in CRA, exhibited an antagonistic correlation with potential colorectal pathogens (such as *Bacteroides*), while also interacting positively with SCFA-producing bacteria like *Prevotella* and *Bifidobacterium.* Notably, archaea play a critical role in directing microbial community metabolism towards optimal SCFA synthesis by consuming bacterial byproducts and preventing metabolite accumulation [63], which is essential for maintaining colon homeostasis. On the functional level, we observed an increase in genes related to methanogenesis and hydrogenotrophic methane pathways, which aligns with the predominance of methanogenic archaea enterotype in HP. The depletion of methanogenesis pathways may be attributed to a reduction in substrates, as we found a positive correlation between SCFA-producing bacteria and methanogen species and pathways. Further experimental studies are needed to understand the relationship between the observed archaea-bacteria interactions and colorectal tumorigenesis. To investigate whether there is a similar interaction between archaea and fungi in the development of CRA, our findings revealed a significant positive correlation between salt-tolerant archaea Haloarchaea, enriched in CRA, and opportunistic pathogenic fungi such as *Alternaria*,* Penicillium*, and *Malassezia.* A salty diet may be a contributing factor to the enrichment of Haloarchaea in CRA patients, and previous reports suggest that salty diets alter SCFA levels in feces. SCFAs are known to prevent colon tumor development [64]. This indicates that the interactions between archaea, bacteria, and fungi play an important role in colorectal tumorigenesis.

Microbiome-related markers can be used to distinguish the stages of CRA [65, 66]. Hua et al. [67] developed a predictive model based on bacterial species. Additionally, studies have shown that the pre-cancerous lesions of colon cancer are associated with changes in the abundance of Firmicutes species [68]. The findings from these studies may partially explain the utility of enterotype in distinguishing CRA from control populations. In this study, we selected the top 10 bacterial, 10 fungal, and 10 archaeal species based on relative abundance and LDA scores to construct diagnostic models. This approach was chosen to focus on the most dominant and characteristic taxa, which are likely to contribute the strongest signal for distinguishing populations. Using these taxa, the combined multi-domain microbial model achieved an AUC of 0.85 in distinguishing HAP from CAP, demonstrating its potential in CRA diagnosis. We recognize that this simplified approach may exclude informative low-abundance taxa and thus limit the generalizability of the model. Future studies could explore including additional taxa to further improve model performance. Among these microbial features, Methanobrevibacter and Prevotella became key features in the model, consistent with our findings. However, despite the important discoveries, there are some limitations in this study. First, we did not assess the correlations among the gut microbiome, clinical parameters, and enterotypes, and therefore cannot establish precise causal relationships between microbial composition and disease. Second, as a cross-sectional study, we were unable to track longitudinal changes in the microbiome, making it difficult to determine the dynamics of gut microbial communities during disease progression. We acknowledge that both genetic and cultural factors may contribute to the observed microbial differences, and our study aims to identify cross-boundary enterotypes rather than establish a direct causal link with altitude [69]. Additionally, to ensure cross-domain consistency, we analyzed microbial composition at the genus level, which may overlook finer diagnostic signals detectable at the species level. Unequal sample distribution among enterotypes may also introduce bias in disease prediction. Furthermore, our predictive models were constructed based on specific populations, limiting their generalizability to other regions or populations. Functional analyses were based on gene abundance–derived pathway predictions without metabolomic or transcriptomic validation, and therefore remain predictive. Environmental factors such as geography, diet, and lifestyle may also confound the observed enterotype distributions and disease associations. Taken together, these limitations suggest that our findings should be interpreted with caution, and that larger, multi-regional longitudinal cohort studies, along with biological validation, are necessary to further explore population-specific microbial markers and to assess the robustness of the predictive models.

## Conclusions

In conclusion, this study comprehensively explored the enterotype variations of bacteria, fungi, and archaea in HP and CP through metagenomic sequencing. We observed differences in the gut microbiome diversity, abundance, and interactions across different regions and disease states. The identification of population and disease-specific intestinal types can help stratify individuals at high risk of colorectal adenoma and potentially guide targeted early screening strategies. Additionally, our findings highlight the diagnostic potential of combining fungal, archaeal, and bacterial features as universal microbial markers for distinguishing HAP from CAP. Furthermore, our results provide predictive models to differentiate HP and HAP, helping to enhance our understanding of the interactions between enterotypes and their impact on CRA development in high-altitude regions, offering new insights into the field.

## Supplementary Information

Supplementary Material 1.

Supplementary Material 2.

Supplementary Material 3.

Supplementary Material 4.

Supplementary Material 5.

Supplementary Material 6.

Supplementary Material 7.

Supplementary Material 8.

Supplementary Material 9.

## Data Availability

The sequence data supporting the findings of this research have been archived in the National Center for Biotechnology Information, with the primary accession number PRJNA1194680 and PRJNA1194747. Here is the link for uploading the raw data: https://www.ncbi.nlm.nih.gov/bioproject/PRJNA1194680 and https://www.ncbi.nlm.nih.gov/bioproject/PRJNA1194747.

## Abbreviations

CRA: Colorectal Adenoma
CP: Coastal population group
HP: High-altitude Population
CAP: Coastal Adenoma Population
HAP: High-altitude Adenoma Population
HCP: High-altitude Control Population
SCFAs: Short-Chain Fatty Acids
PAM: Partitioning Around Medoids
IL-1β: Interleukin-1β
IL-6: Interleukin-6
COX-2: Cyclooxygenase-2
IL-10: Interleukin 10
VEGF: Vascular Endothelial Growth Factor
PCA: Principal Component Analysis
LDA: Linear discriminant analysis
