# Supplementary material for: The study on the identification of cross-boundary microbiome enterotypes between high-altitude and coastal populations and their predictive value
Source: BMC Microbiol. 2026 Jan 29;26:225. doi: 10.1186/s12866-025-04578-0 (PMC12973879; doi:10.1186/s12866-025-04578-0)
Supplement: Supplementary file 1 — Supplementary Material 1. [file 12866_2025_4578_MOESM1_ESM.docx]

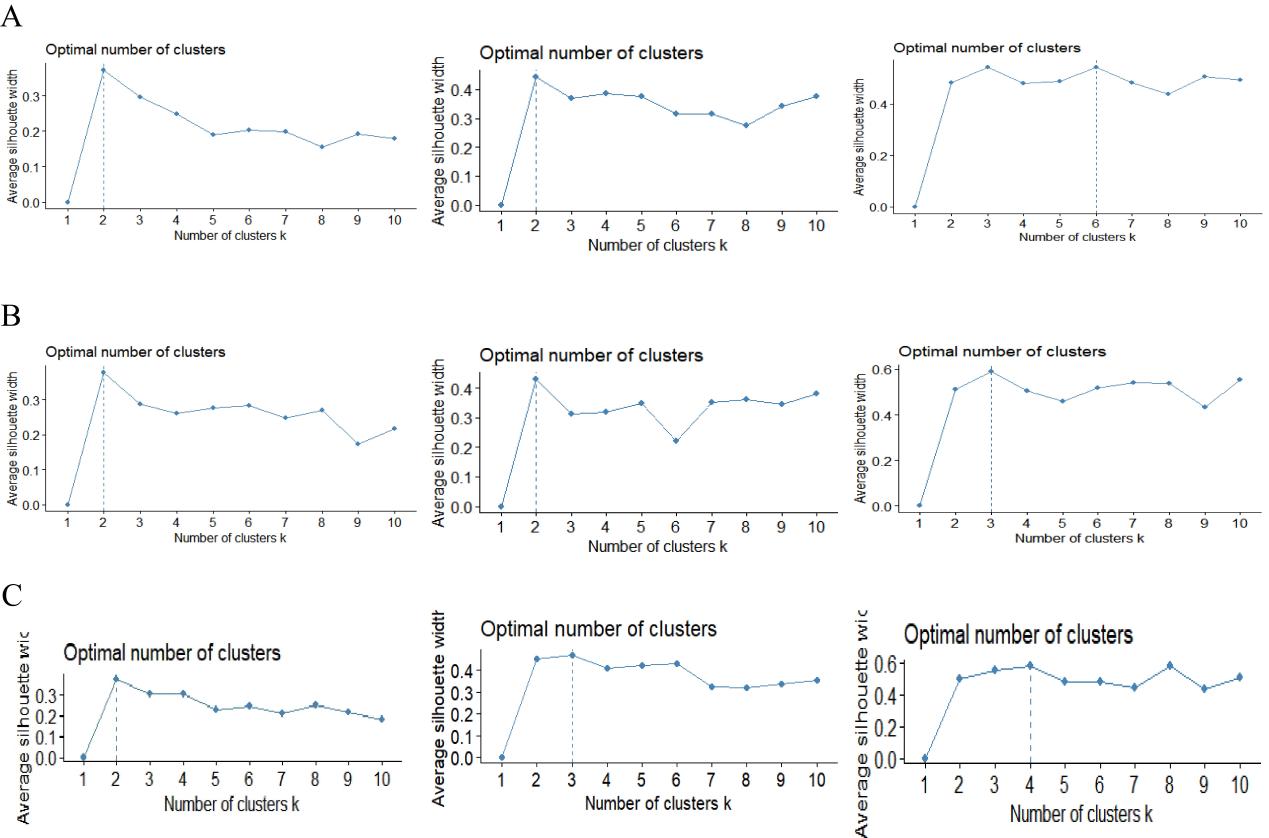


**Fig. S1** A: Calinski-Harabasz (CH) index analysis based on Jensen Shannon divergence (JSD) distance in bacterial enterotypes. The optimal number of clusters was determined when K was 2, 2 and 6, respectively. B: CH index analysis based on JSD distance in fungal intestinal type. When K was 2, 2 and 3, the corresponding clusters were determined to be the optimal number of clusters. C: CH index analysis based on JSD distance in archaeal gut types. The corresponding clusters were determined to be the optimal number of clusters when K was 2, 3, and 4, respectively.


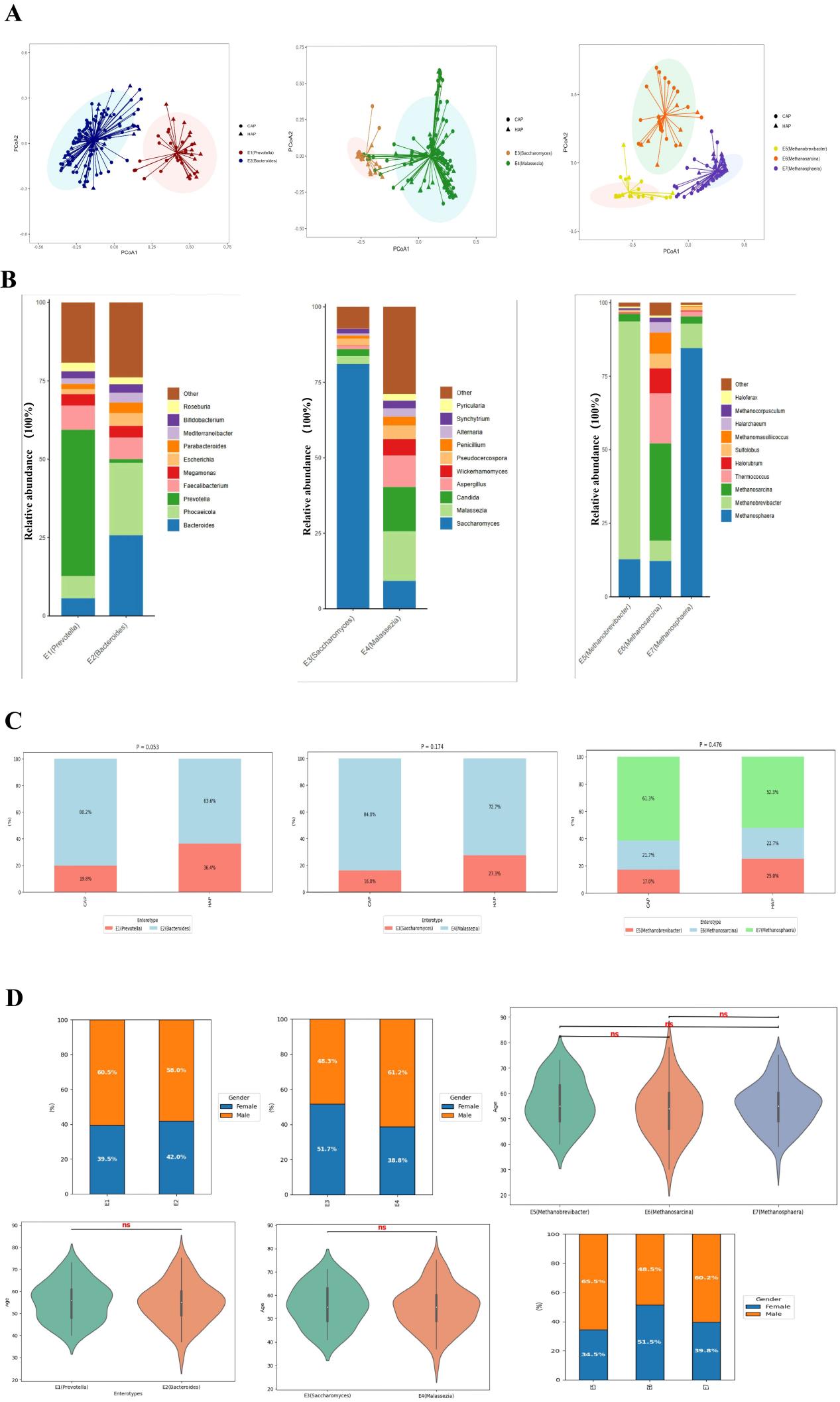


**Fig. S2** Enterotype analysis of transboundary microorganisms in 150 samples. A: The results of enterotype analysis were visualized using PCoA based on the Bray -- Curtis distance of genus composition. B: The proportion of microbial genera in each intestinal type. C: The proportion of transboundary microbial intestinal types in the CAP and HAP groups. D: Distribution of clinical factors such as sex and age in intestinal patterns. ns indicated no statistical difference between the two groups (P>0.05).


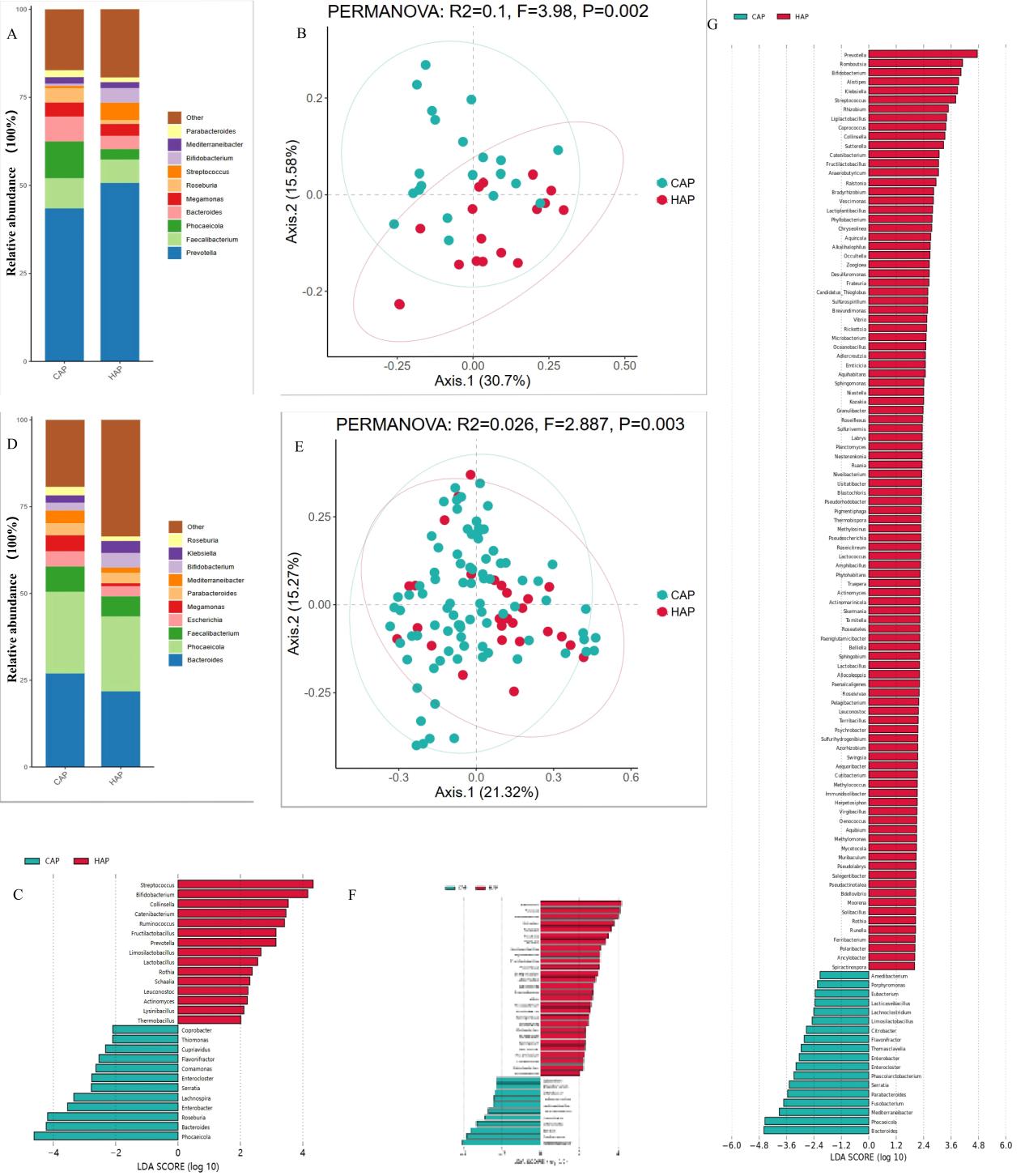


**Fig. S3** Different bacterial compositions of E1 and E2 enterotype samples from CAP and HAP.

A: Abundance of gut microbiota communities at the genus level for different bacterial compositions of E1 enterotype samples. B: PCA plot of the E1 enterotype sample visualizing two human cohorts. Red and blue dots represent CAP and HAP, respectively. C: E1 enterotype samples were found to have differentially abundant bacterial genera in CAP and HAP samples by LDA. D: Abundance of gut microbiota communities at the genus level for the different bacterial composition of the E2 enterotype samples. E: PCA plot of the E2 enterotype sample visualizing two human cohorts. F: E2 enterotype samples were found to have differentially abundant bacterial genera in CAP and HAP samples by LDA. G: Differences in bacterial gut microbiota characteristics between CAP and HAP based on all samples.


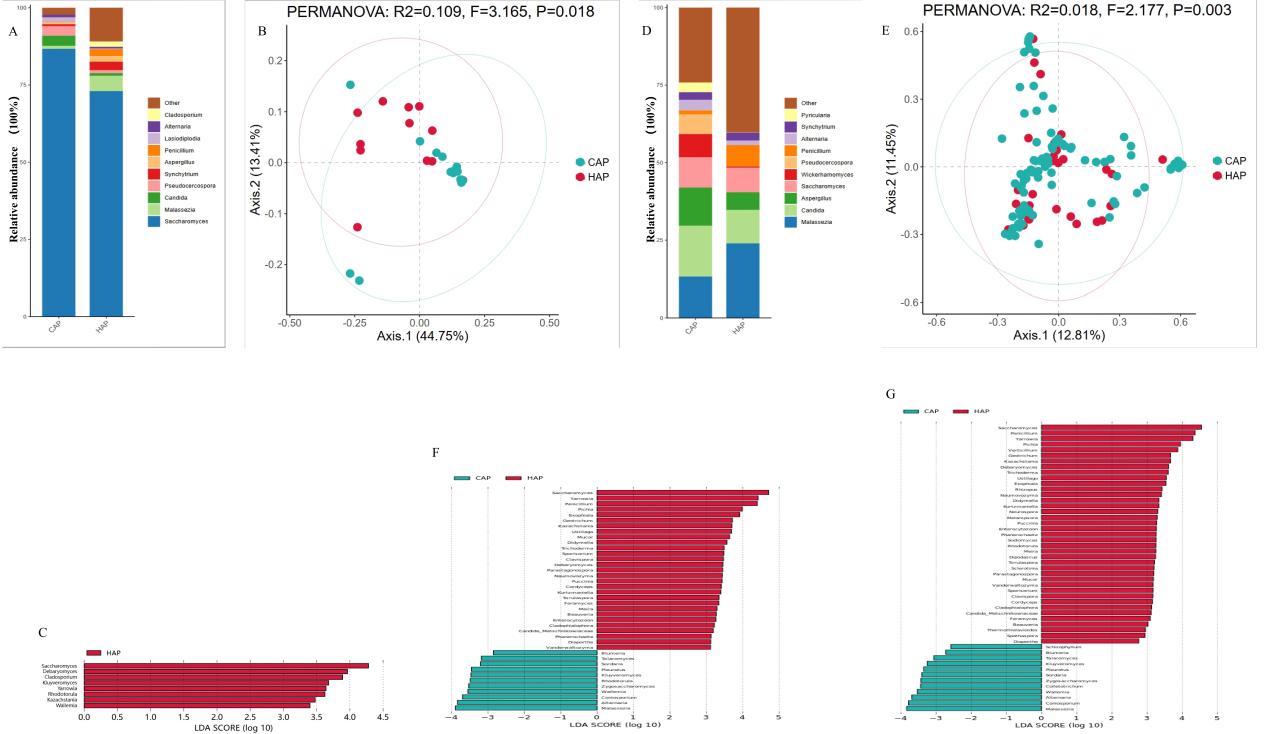


**Fig. S4** Different fungal compositions of E3 and E4 enterotype samples from CAP and HAP. A: Gut microbiota community abundance at the genus level for different fungal compositions of E3 enterotype samples. B: PCA plot of the E3 enterotype sample visualizing two human cohorts. Red and blue dots represent CAP and HAP, respectively. C: E3 enterotype samples were found to have differentially abundant fungal genera in CAP and HAP samples by LDA. D: Abundance of gut microbiota communities at the genus level for different fungal compositions in the E4 enterotype sample. E: PCA plot of the E4 enterotype sample visualizing two human cohorts. F: E4 enterotype samples were found to have differentially abundant fungal genera in CAP and HAP samples by LDA. G: Differences in fungal gut microbiota characteristics between CAP and HAP based on all samples.


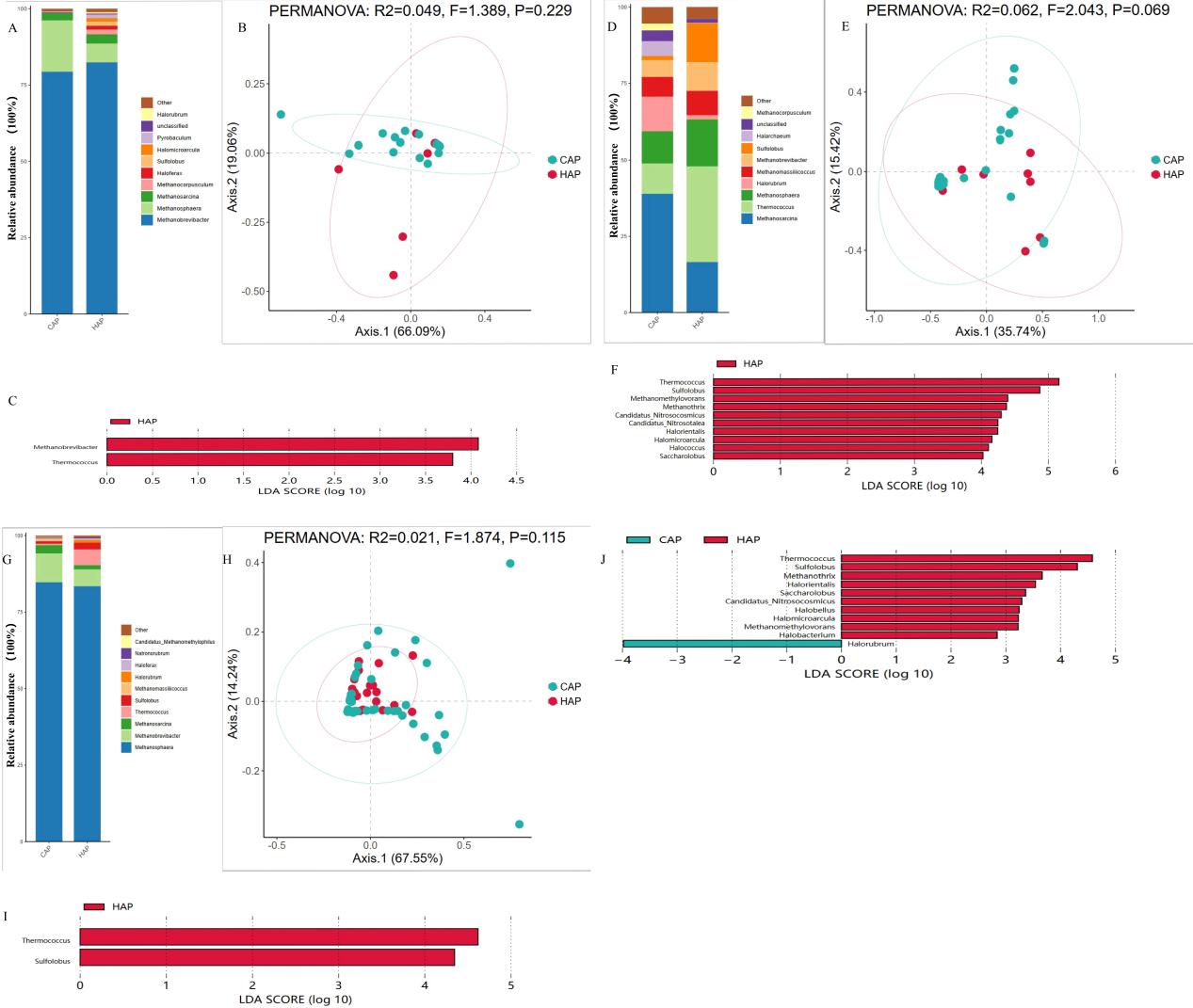


**Fig. S5** Different archaeal composition of E5, E6, and E7 enterotype samples from CAP and HAP. A: Abundance of gut microbiota communities at the genus level for different archaeal compositions of E5 enterotype samples. B: PCA plot of E5 enterotype samples visualizing two human cohorts. Red and blue dots represent CAP and HAP, respectively. C: E5 enterotype samples were found to have differentially abundant archaeal genera in CAP and HAP samples by LDA. D: Abundance of gut microbiota communities at the genus level for the different archaeal composition of E6 intestinal type samples. E: PCA plot of the E6 enterotype sample visualizing two human cohorts. F: E6 enterotype samples were found to have differentially abundant archaeal genera in CAP and HAP samples by LDA. G: Abundance of gut microbiota communities at the genus level for the different archaeal composition of E7 enterotype samples. H: PCA plot of E7 enterotype samples visualizing two human cohorts. I: E7 enterotype samples identified differentially abundant archaeal genera in CAP and HAP samples by LDA. J: Differences in archaeal gut microbiota characteristics between CAP and HAP based on all samples.


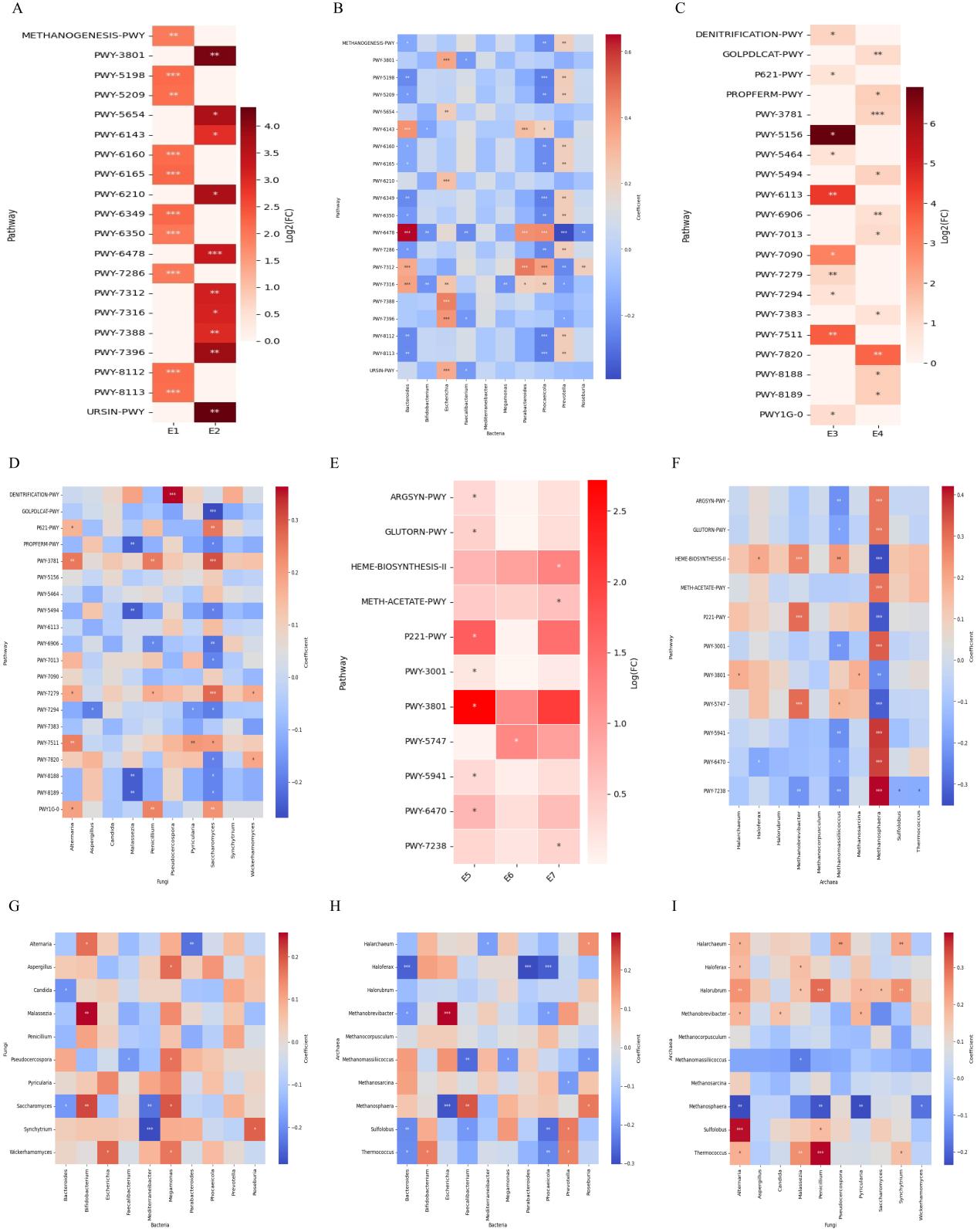


**Fig. S6** Metabolic pathways associated with enterotypes of transkingdom microbes. A-B: Bacterial pathways enriched in different bacterial enterotypes (A) and related bacterial genera (B). C-D: Bacterial pathways enriched in different fungal intestinal types (C) and related fungal genera (D). E-F: Bacterial pathways enriched in different archaeal intestinal types (E) and associated individual bacterial genera (F). G: correlation between fungal and bacterial enterotypes in CAP and HAP. H: correlation between archaeal and bacterial enterotypes in CAP and HAP. I: correlation between archaeal and fungal enterotypes in CAP and HAP. Log(FC) represents the log-transformed fold change of the mean relative abundance of the pathway relative to the other pathways in each transkingdom microbial intestinal type. Asterisks indicate the statistical significance of the multiple testing corrected Pearson correlation test (top) and the multiple testing corrected Wilcoxon rank-sum test (bottom) : * adjusted p<0.05, ** adjusted p<0.01, and *** adjusted p<0.001.


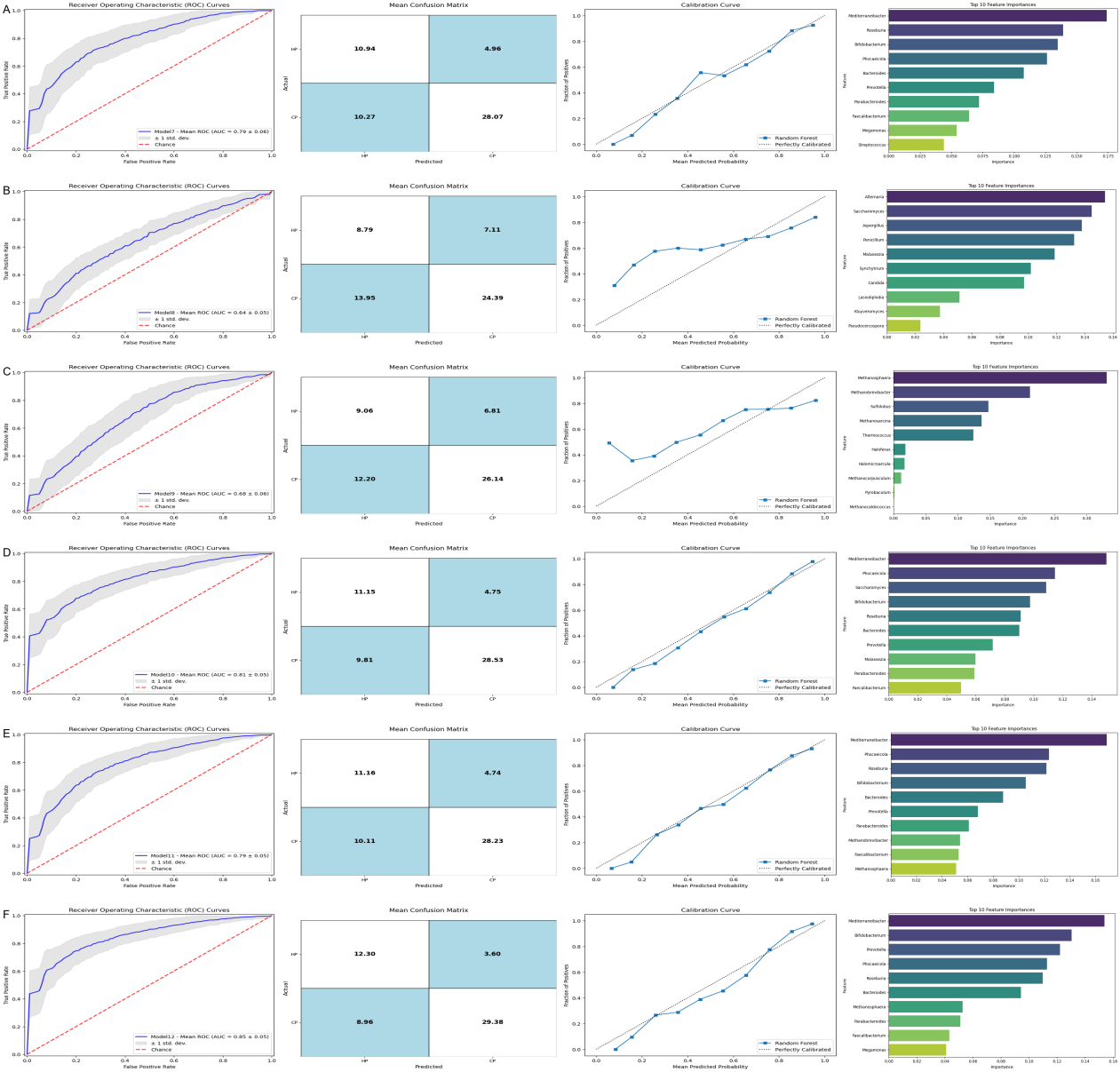


**Fig. S7** A: Prediction model based on the 10 bacteria enriched in CAP and HAP. The average ROC curve shows the performance of the prediction model, the average confusion matrix shows the classification performance of the model, the calibration curve shows the agreement between the predicted probability and the actual results, and the bar graph shows the average feature importance of the 10 bacterial species used in the prediction model. B: Prediction model based on 10 fungi enriched in CAP and HAP. C: Prediction model based on 10 archaeal species enriched in CAP and HAP. D: Diagnostic performance of the model combining bacterial and fungal microbial markers to distinguish CAP from HAP. E: Diagnostic performance of the model combining bacterial and archaeal microbial markers to distinguish CAP from HAP. F: Diagnostic performance of a model incorporating bacterial, fungal, and archaeal microbial markers to distinguish CAP from HAP.


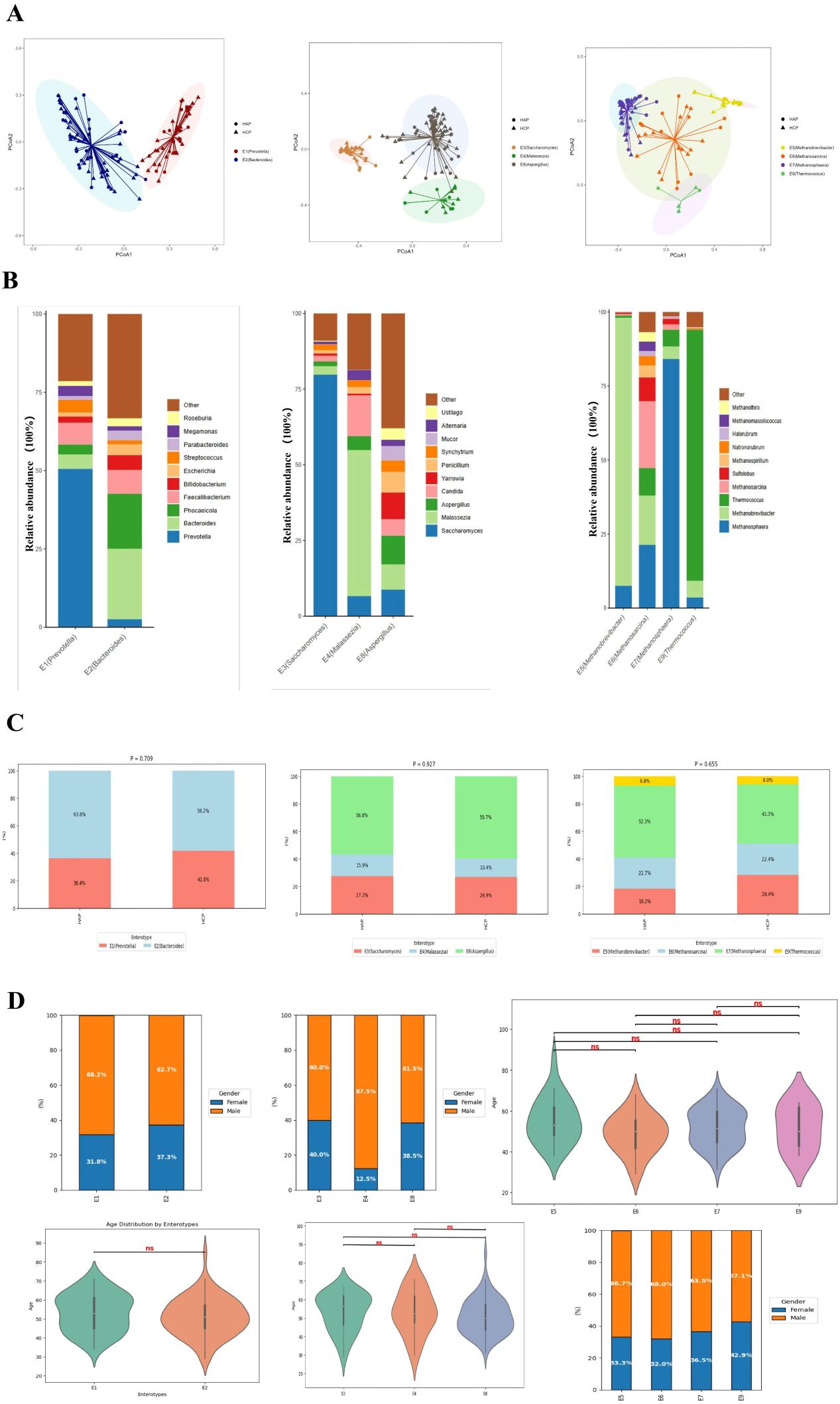


**Fig. S8** Analysis of intestinal intestinal types of transboundary microorganisms in 112 samples. A: The results of enterotype analysis were visualized using PCoA based on the Bray -- Curtis distance of genus composition. B: The proportion of microbial genera in each intestinal type. C: The proportion of transboundary microbial intestinal types in HAP and HCP groups. D: Distribution of clinical factors such as sex and age in intestinal patterns. ns indicated no statistical difference between the two groups (P>0.05).


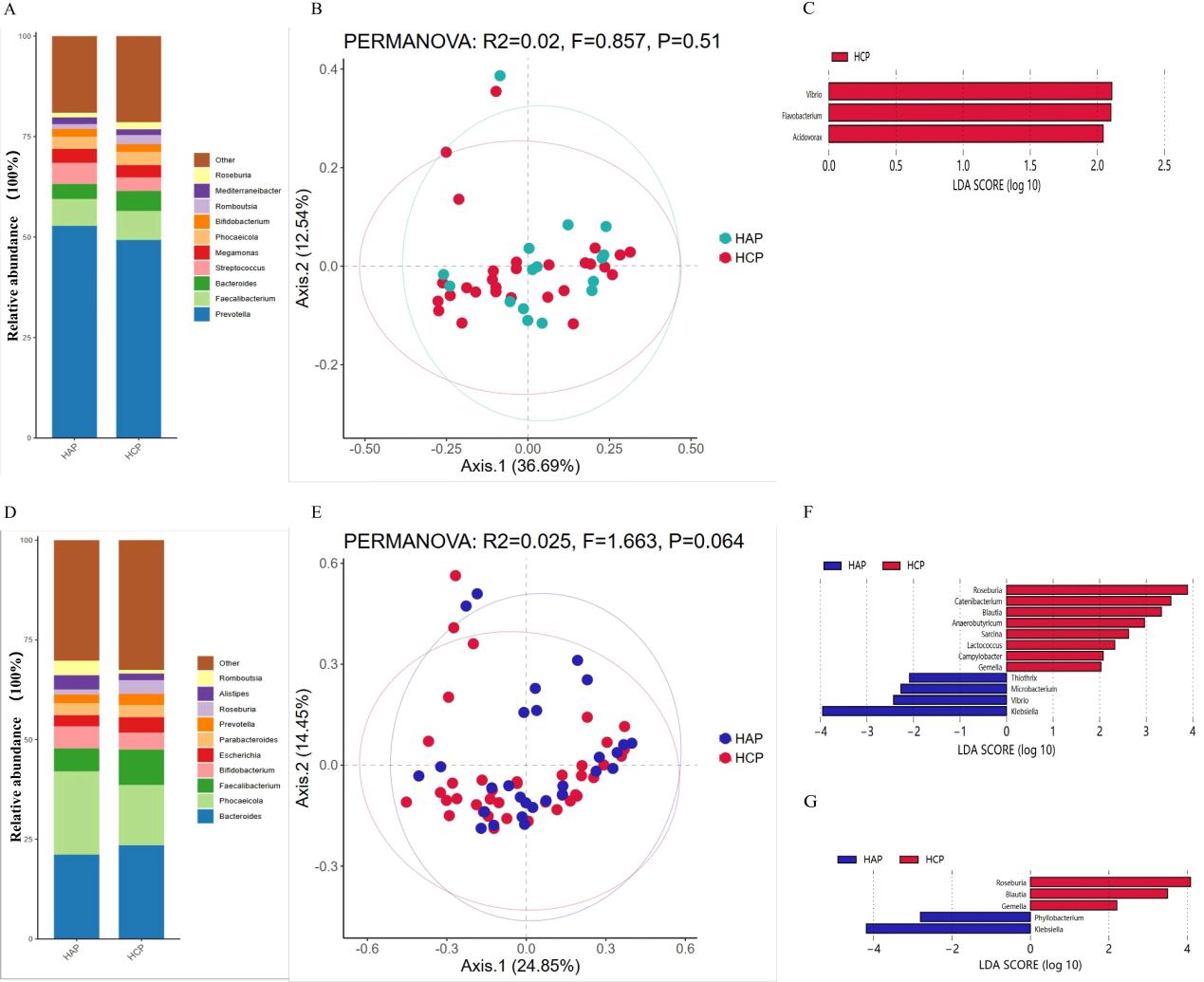


**Fig. S9** Different bacterial compositions of E1 and E2 enterotype samples from HAP and HCP. A: Abundance of gut microbiota communities at the genus level for different bacterial compositions of E1 enterotype samples. B: PCA plot of the E1 enterotype sample visualizing two human cohorts. Red and blue dots represent HAP and HCP, respectively. C: E1 enterotype samples were found to have differentially abundant bacterial genera in HAP and HCP samples by LDA. D: Abundance of gut microbiota communities at the genus level for the different bacterial composition of the E2 enterotype samples. E: PCA plot of the E2 enterotype sample visualizing two human cohorts. F: E2 enterotype samples were found to have differentially abundant bacterial genera in HAP and HCP samples by LDA. G: Differences in bacterial gut microbiota characteristics between HAP and HCP based on all samples.


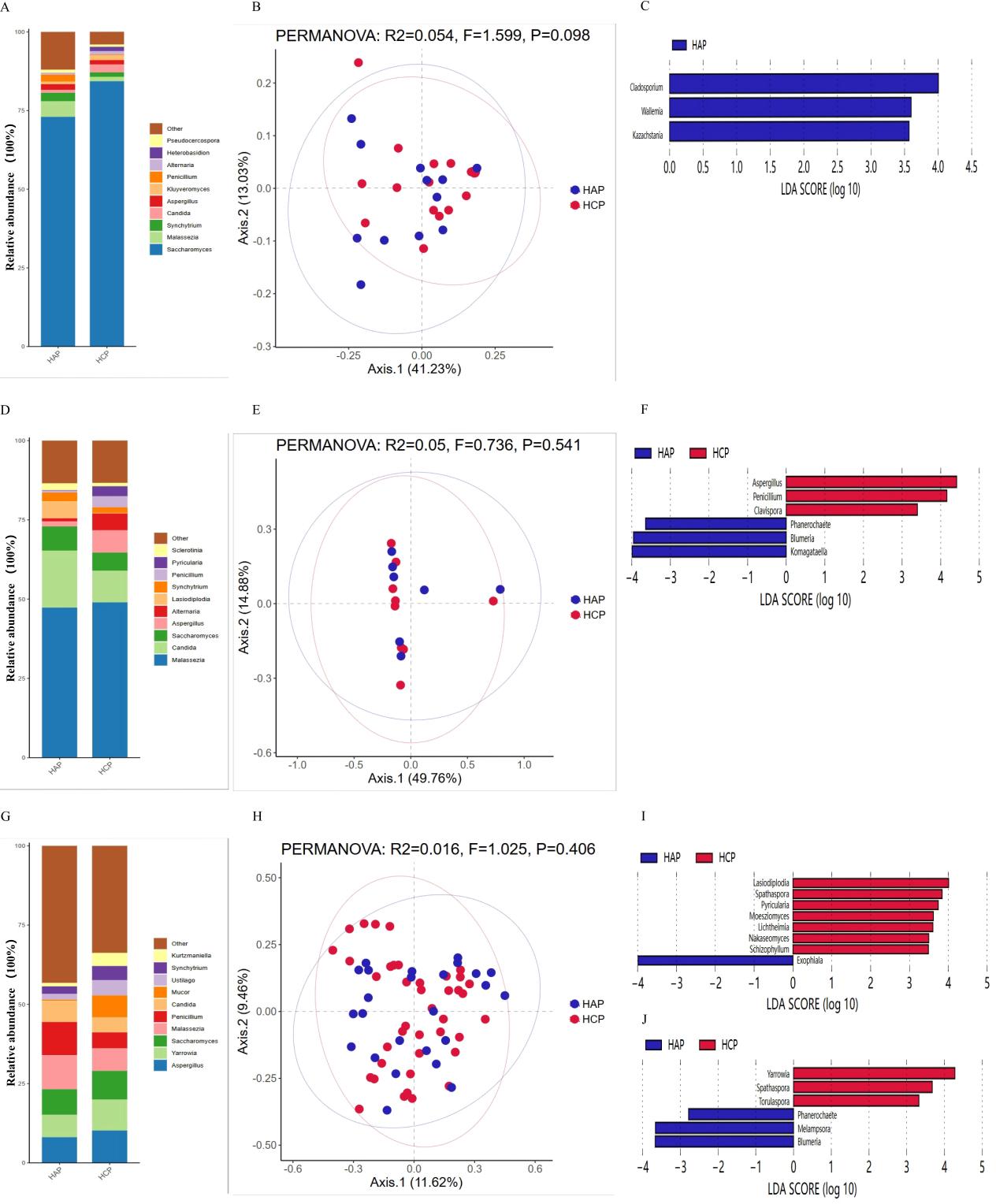


**Fig. S10** Different fungal compositions of E3, E4, and E8 enterotype samples from HAP and HCP. A: Gut microbiota community abundance at the genus level for different fungal compositions of E3 enterotype samples. B: PCA plot of the E3 enterotype sample visualizing two human cohorts. Red and blue dots represent HAP and HCP, respectively. C: E3 enterotype samples were found to have differentially abundant fungal genera in HAP and HCP samples by LDA. D: Abundance of gut microbiota communities at the genus level for different fungal compositions in the E4 enterotype sample. E: PCA plot of the E4 enterotype sample visualizing two human cohorts. F: E4 enterotype samples were found to have differentially abundant fungal genera in HAP and HCP samples by LDA. G: Gut microbiota community abundance at the genus level for different fungal compositions of E8 intestinal type samples. H: PCA plot of E8 enterotype samples visualises two human cohorts. I: E8 enterotype samples were found to have differentially abundant fungal genera in HAP and HCP samples by LDA. J: Differences in fungal gut microbiota characteristics between HAP and HCP based on all samples.


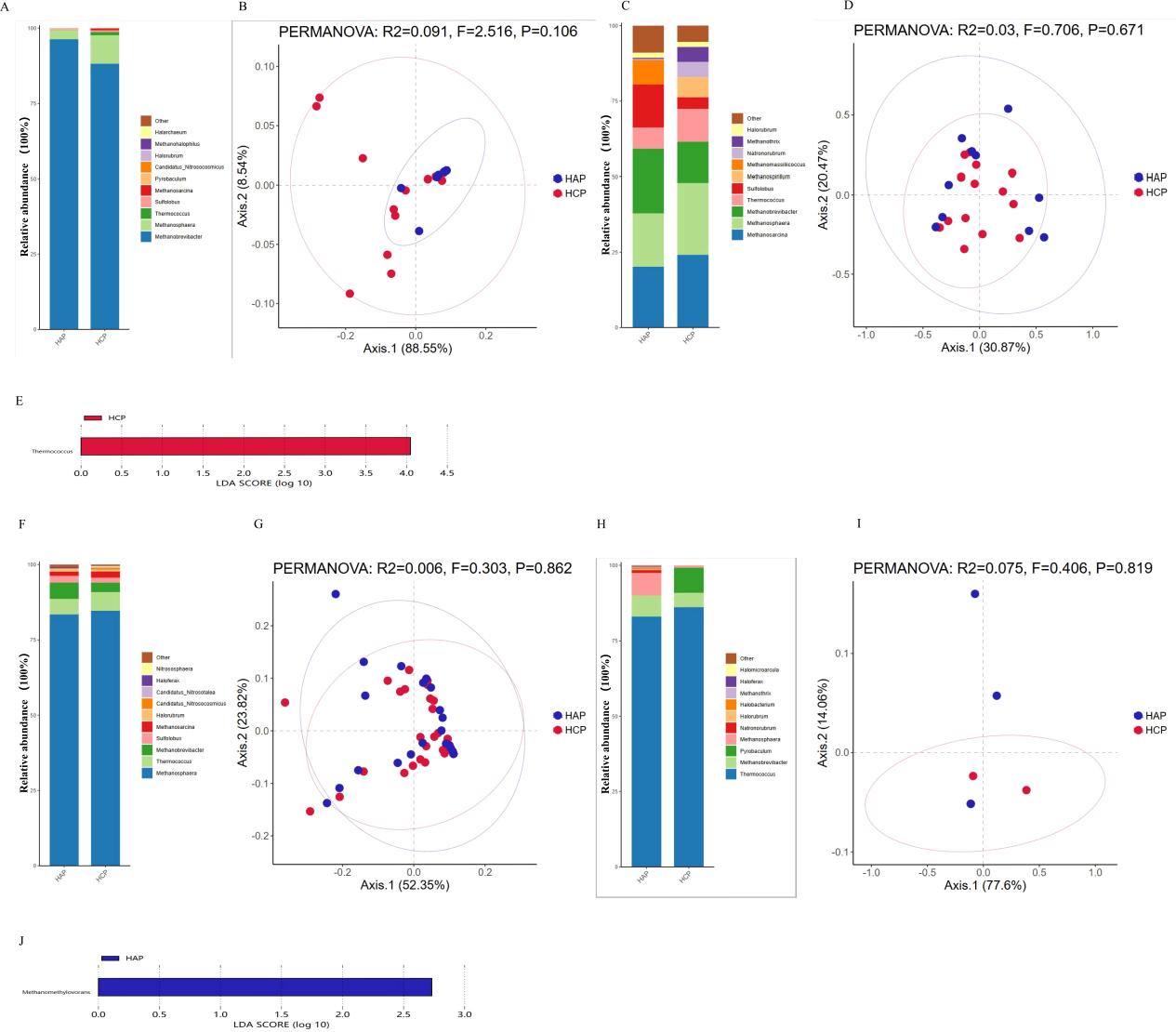


**Fig. S11** Different archaeal composition of E5, E6, E7, and E9 enterotype samples from HAP and HCP. A: Abundance of gut microbiota communities at the genus level for different archaeal compositions of E5 enterotype samples. B: PCA plot of E5 enterotype samples visualizing two human cohorts. Red and blue dots represent HAP and HCP, respectively. C: E5 enterotype samples were found to have differentially abundant archaeal genera in HAP and HCP samples by LDA. D: Abundance of gut microbiota communities at the genus level for the different archaeal composition of E6 intestinal type samples. E: PCA plot of the E6 enterotype sample visualizing two human cohorts. F: Abundance of gut microbiota communities at the genus level for the different archaeal composition of E7 enterotype samples. G: Abundance of gut microbiota communities at the genus level for the different archaeal composition of E7 enterotype samples. H: Gut microbiota community abundance at the genus level for different archaeal composition of E9 intestinal type samples. I: PCA plot of E9 intestinal type samples visualises two human cohorts. J: Differences in archaeal gut microbiota characteristics between HAP and HCP based on all samples.


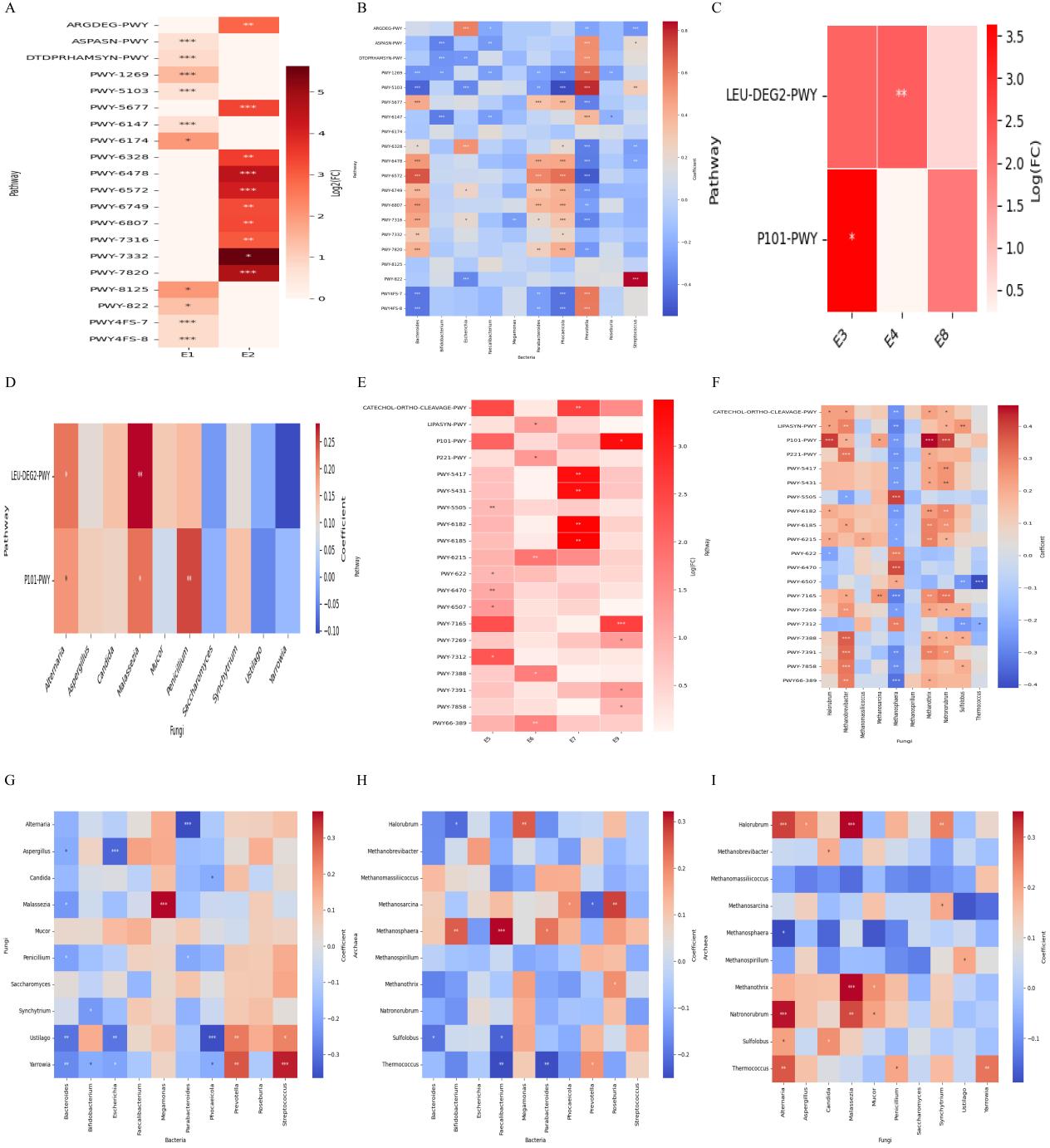


**Fig. S12** Metabolic Pathways associated with Enterotypes of transkingdom microbes. A-B: Bacterial pathways enriched in different bacterial enterotypes (A) and related bacterial genera (B). C-D: Bacterial pathways enriched in different fungal intestinal types (C) and related fungal genera (D). E-F: Bacterial pathways enriched in different archaeal intestinal types (E) and associated individual bacterial genera (F). G: correlation between fungal and bacterial enterotypes in HAP and HCP. H: correlation between archaeal and bacterial enterotypes in HAP and HCP. I: Correlation between archaeal and fungal enterotypes in HAP and HCP. Log(FC) represents the log-transformed fold change of the mean relative abundance of the pathway relative to the other pathways in each transkingdom microbial intestinal type. Asterisks indicate the statistical significance of the multiple testing corrected Pearson correlation test (top) and the multiple testing corrected Wilcoxon rank-sum test (bottom) : * adjusted p<0.05, ** adjusted p<0.01, and *** adjusted p<0.001.


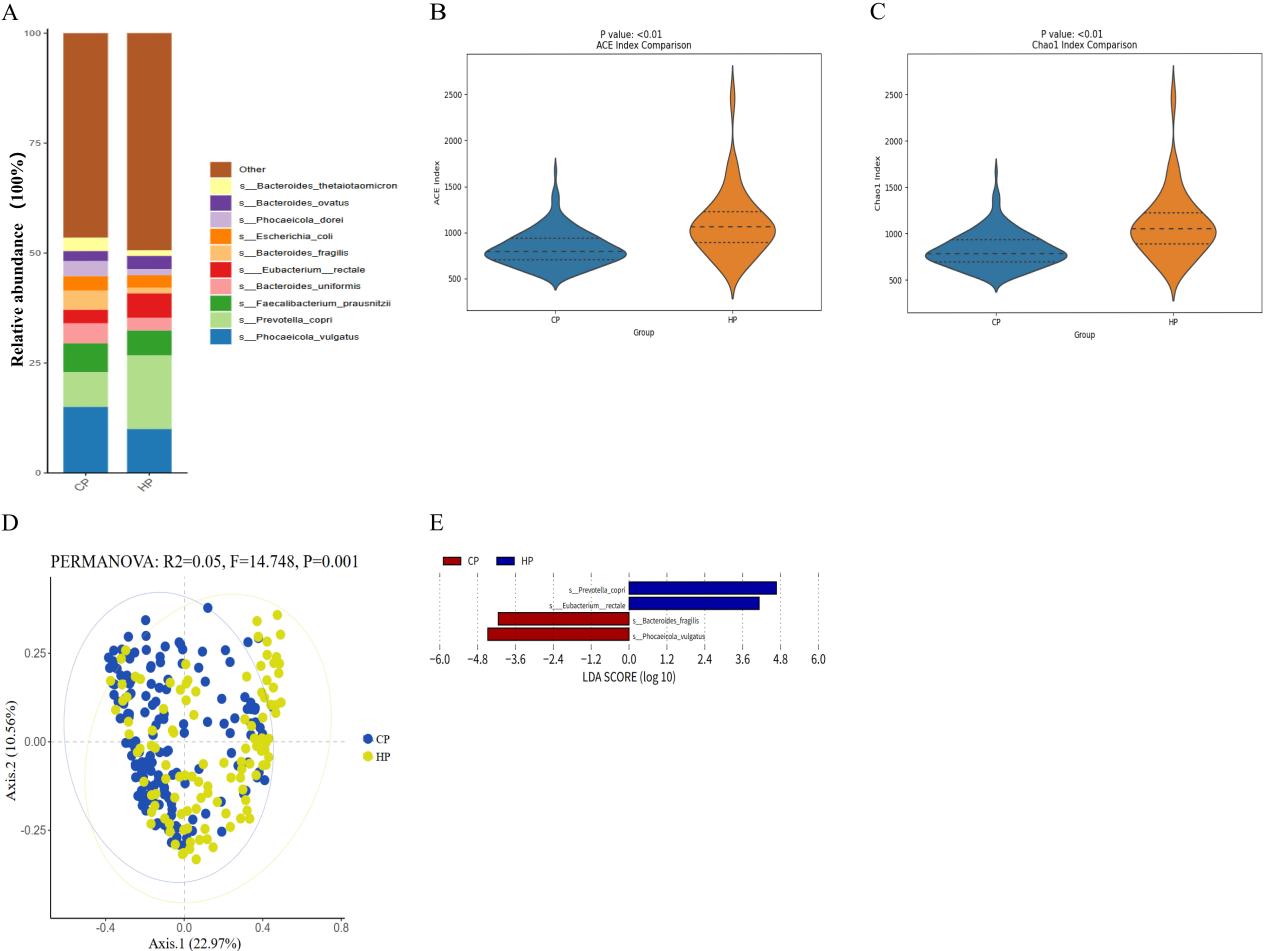


**Fig. S13** Gut microbiota diversity, composition, and differential species between CP and HP groups. A: Relative abundances of dominant species are shown in bar plots. B-C: Alpha diversity was assessed using the ACE and Chao1 indices to evaluate community richness, showing statistically significant differences between the two groups. D: Beta diversity was analyzed by principal coordinates analysis (PCoA) based on Bray–Curtis distances, revealing distinct clustering of samples. E: Differential species between CP and HP groups were further identified using LEfSe analysis, highlighting taxa enriched in each group.
