# Supplementary material for: The study on the identification of cross-boundary microbiome enterotypes between high-altitude and coastal populations and their predictive value
Source: BMC Microbiol. 2026 Jan 29;26:225. doi: 10.1186/s12866-025-04578-0 (PMC12973879; doi:10.1186/s12866-025-04578-0)
Supplement: Supplementary file 5 — Supplementary Material 5. [file 12866_2025_4578_MOESM5_ESM.docx]

**Table.** The relationship among age, gender and different groups.

|  | CP group (n=184) | HP group (n=111) | P value |
| --- | --- | --- | --- |
| Age (year, mean ± SD) | 53.67±9.28 | 51.76±10.08 | 0.098 |
| Gender, N% |  |  | 0.069 |
| Male | 91 (49.5) | 67 (60.4) |  |
| Female | 93 (50.5) | 44 (39.6) |  |

|  | CAP group (n=106) | HAP group (n=43) | P value |
| --- | --- | --- | --- |
| Age (year, mean ± SD) | 55.64±8.91 | 52.81±8.73 | 0.080 |
| Gender, N% |  |  | 0.628 |
| Male | 62 (58.5) | 27 (62.8) |  |
| Female | 44 (41.5) | 16 (37.2) |  |

|  | HCP group (n=68) | HAP group (n=43) | P value |
| --- | --- | --- | --- |
| Age (year, mean ± SD) | 51.09±10.85 | 52.81±8.73 | 0.382 |
| Gender, N% |  |  | 0.677 |
| Male | 40 (58.8) | 27 (62.8) |  |
| Female | 28 (41.2) | 16 (37.2) |  |
