# Supplementary material for: The study on the identification of cross-boundary microbiome enterotypes between high-altitude and coastal populations and their predictive value
Source: BMC Microbiol. 2026 Jan 29;26:225. doi: 10.1186/s12866-025-04578-0 (PMC12973879; doi:10.1186/s12866-025-04578-0)
Supplement: Supplementary file 8 — Supplementary Material 8. [file 12866_2025_4578_MOESM8_ESM.docx]

**SUPPLEMENTARY TEXT 1**

**Results**

**Overall characteristics of two bacterial enterotype microbiota in coastal and high-altitude adenoma populations**

First, PCoA analysis was performed on CAP and HAP (Figure S2A). Then, JSD distance metric was used to cluster 150 samples based on the relative abundance of bacterial genera at the genus level. The corresponding clusters were determined by considering the silhouette width, CH index, DBI index, and Dunn index when K was set to 2, 2, and 3, respectively (Figure S1B). Two bacterial enterotypes were defined as Prevotella (E1, n=37) and Bacteroides (E2, n=113) based on the dominant genera in each group. Two fungal enterotypes were assigned to Saccharomyces (E3, n=29) and Malassezia (E4, n=121). Three archaeal enterotypes were defined as Methanobrevibacter (E5, n=29), Methanosarcina (E6, n=33), and Methanosphaera (E7, n=88). The relative abundance of bacterial (cross-domain microbiota) genera in each enterotype is shown in Figure S2B. Briefly, *Prevotella* (41.78%) and *Faecalibacterium* (6.86%) are relatively abundant in the E1 enterotype. *Bacteroides* (23.41%) and *Phocaeicola* (21.00%) are relatively abundant in the E2 enterotype. Saccharomyces (81.07%) is relatively abundant in the E3 enterotype. *Malassezia* (16.38%) and *Candida* (14.72%) are relatively abundant in the E4 enterotype. *Methanobrevibacter* (80.52%) is predominant in the E5 enterotype. *Methanosarcina* (32.17%) and *Thermococcus* (16.44%) are more abundant in the E6 enterotype. *Methanosphaera* (84.40%) is the dominant genus in the E7 enterotype. Furthermore, we analyzed the proportions of cross-domain microbiota enterotypes in the CAP and HAP populations, revealing that the E1 enterotype was more prevalent in HAP (36.4% vs. 19.8%), the E3 enterotype in fungi was more common in HAP (27.3% vs. 16.0%), and the E5 enterotype in archaea was predominant in HAP (25.0% vs. 17.0%) (Figure S2C). Additionally, we analyzed the distribution of clinical factors such as age and sex among the cross-domain microbiota enterotypes. The results showed no statistically significant differences in clinical factors between the enterotypes (P>0.05) (Figure S2D).

**Bacterial composition at the genus level in the E1 enterotype of coastal and high-altitude populations**

In the E1 enterotype, the bacterial composition at the genus level in the two populations is shown in Figure S3A. The five dominant genera in both populations were identified as *Prevotella, Faecalibacterium, Phocaeicola, Bacteroides,* and *Megamonas*. PCA analysis showed significant differences in microbial composition between the two populations (P=0.002) (Figure S3B). Further analysis using LDA identified 27 genera that significantly differed between CAP and HAP (Figure S3C). Of these, 15 genera, including *Streptococcus, Bifidobacterium,* and *Collinsella,* were significantly enriched in the HAP group; 12 genera, including *Phocaeicola, Bacteroides,* and *Roseburia,* were significantly enriched in the CAP group.

**Bacterial composition at the genus level in the E2 enterotype of coastal and high-altitude populations**

In the E2 enterotype, the relative abundance of bacterial genera in the two populations is shown in Figure S3D. The five major bacterial genera in both populations were *Bacteroides, Phocaeicola, Faecalibacterium, Escherichia,* and *Megamonas.* PCA analysis revealed significant separation between the two groups (P=0.003) (Figure S3E). Additionally, LDA was applied to identify specific genera for each group (Figure S3F). In summary, 11 genera were significantly enriched in the CAP group, including *Mediterraneibacter, Fusobacterium,* and *Serratia*; 28 genera were identified in the HAP group, including *Romboutsia, Alistipes,* and *Bifidobacterium*. To verify the reliability of the enterotype classification criteria, we also analyzed samples from populations without enterotype classification. The results showed differences in 119 genera between the two groups (Figure S3G). Specifically, 101 bacterial genera (including *Prevotella, Romboutsia,* and *Bifidobacterium*) were significantly enriched in the HAP group, while 18 biomarkers (including *Bacteroides, Phocaeicola,* and *Mediterraneibacter*) were most abundant in the CAP group.

**Fungal composition at the genus level in the E3 enterotype of coastal and high-altitude populations**

In the E3 enterotype, the fungal composition at the genus level in the two populations is shown in Figure S4A. The five dominant genera were *Saccharomyces, Malassezia, Candida, Pseudocercospora,* and *Synchytrium.* PCA analysis revealed significant differences in microbial composition between the two groups (P<0.05) (Figure S4B). Further LDA analysis identified 8 genera that differed between CAP and HAP (Figure S4C). Among these, *Malassezia, Debaryomyces,* and *Cladosporium* were significantly enriched in the HAP group.

**Fungal composition at the genus level in the E4 enterotype of coastal and high-altitude populations**

In the E4 enterotype, the relative abundance of fungal genera in the two populations is shown in Figure S4D. The five major fungal genera in both populations were *Malassezia, Candida, Aspergillus, Saccharomyces,* and *Wickerhamomyces.* PCA analysis indicated significant separation between the two groups (P=0.003) (Figure S4E). LDA analysis identified specific genera for each group (Figure S4F). In summary, 11 genera were significantly enriched in the CAP group, including *Heterobasidion, Alternaria,* and *Coniosporium;* 11 genera were predominant in the HAP group, including *Malassezia, Yarrowia,* and *Penicillium.* To verify the reliability of the enterotype classification, we also analyzed all samples from different regional populations without enterotype classification. The results showed differences in 51 genera between the two groups (Figure S4G). Specifically, 39 fungal genera (including *Malassezia, Penicillium,* and *Yarrowia*) were significantly enriched in the HAP group, while 12 biomarkers (including *Heterobasidion, Coniosporium,* and *Alternaria*) were most abundant in the CAP group.

**The composition of the gut microbiome at the genus level in the populations of the two regions in the E5 enterotype**

In the E5 enterotype, the genus-level composition of archaea in the two regional populations is shown in Figure S5A. In both populations, the five dominant genera are *Methanobrevibacter, Methanosphaera, Methanosarcina, Methanocorpusculum,* and *Haloferax.* The PCA plot indicates no significant difference in microbial composition between the two populations (P=0.229) (Figure S5B). Further analysis using LDA to identify differences in microbial species between the two groups revealed that two genera, *Thermococcus* and *Sulfolobus*, were significantly enriched in the HAP group (Figure S5C).

**The composition of the gut microbiome at the genus level in the two regions of the E6 enterotype population**

In the E6 enterotype, Figure S5D shows the relative abundance of archaea at the genus level in the two regional populations. In both populations, the five major genera are *Methanosarcina, Thermococcus, Methanosphaera, Halorubrum,* and *Methanomassiliicoccus*. The PCA plot indicates no significant separation between the two groups (P=0.069) (Figure S5E). Additionally, LDA was used to identify specific genera in each group (Figure S5F). In summary, ten genera, including T*hermococcus, Sulfolobus,* and *Methanomethylovorans,* were significantly enriched in the HAP group.

**The composition of the gut microbiome at the genus level in the two regions of the E7 enterotype population**

In the E7 enterotype, Figure S5G shows the relative abundance of archaea at the genus level in the two regional populations. In both populations, the five major genera are *Methanosphaera, Methanobrevibacter, Methanosarcina, Thermococcus,* and *Sulfolobus.* The PCA plot indicates no significant separation between the two groups (P=0.115) (Figure S5H). Additionally, LDA was used to identify specific genera in each group (Figure S5I). In summary, two genera, *Thermococcus* and *Sulfolobus*, were significantly enriched in the HAP group. To validate the reliability of the three enterotype classification standards, we also analyzed all the samples from different regional populations without classifying them by enterotype. The results showed that there were differences in 11 intestinal genera between the two groups (Figure S5J). In summary, 10 bacterial genera, including *Thermococcus, Sulfolobus,* and *Methanothrix*, were significantly enriched in the HAP group, while one biomarker, *Halorubrum*, had the highest abundance in the CAP group.

**Functional differences in enterotypes of different transkingdom microbes**

To characterize the bioactivity potential and the underlying metabolic mechanisms of bacterial enterotypes, we utilized the MetaCyc gene function annotation database to predict the metabolic functions of gut microbiota across different enterotypes in two populations (Figure S6A). In enterotype E1, we observed pathways closely related to the synthesis of aromatic amino acids in archaea (PWY-6160, P < 0.05). Additionally, bacterial contribution pathways in E1 enterotype also participated in the synthesis of archaeal inositol, a crucial component of archaeal cell membranes that plays a role in membrane stability and structural maintenance (PWY-6350), as well as in archaeal protein synthesis (PWY-7286). Furthermore, dominant species in E1 enterotype, such as *Bacteroides, Prevotella,* and *Phocaeicola,* were significantly associated with GLUCOSE1PMETAB-PWY (Figure S6B). In enterotype E3, a close association with the PWY-7279 metabolic pathway was observed (P < 0.05, Figure S6C). This pathway is primarily involved in aerobic respiration and plays a key role in adapting to hypoxic environments, optimizing energy metabolism, and enhancing oxygen utilization efficiency. Moreover, the dominant species in E3 enterotype, *Saccharomyces*, was significantly associated with the degradation of methylphosphonic acid (Figure S6D). In enterotype E5, a significant correlation with the metabolic pathway of octane degradation (P221-PWY, P < 0.05) was observed (Figure S6E), which allows microorganisms to utilize it as a carbon source. Although octane oxidation primarily occurs in certain bacteria and archaea, high-altitude populations may indirectly benefit from gut microbial activities that utilize hydrocarbons or other organic compounds as energy sources, influencing the overall metabolism of high-altitude populations, especially under energy-scarce environmental conditions. Additionally, the dominant species of Methanobrevibacter in E5 enterotype showed a significant positive correlation with the P221-PWY pathway (Figure S6F). This may be closely related to the dominance of cross-boundary microbial enterotypes in high-altitude populations. The close relationship between bacteria and archaea in these populations can be explained by complementary metabolic pathways that coexist in extreme environments. Bacteria support archaea's survival by producing hydrogen, providing intermediate products, and maintaining energy balance, while archaea assist bacteria in energy acquisition through unique metabolic pathways such as methane production and coenzyme synthesis. This symbiotic and interdependent relationship is key to the stability of the gut microbiota in high-altitude populations. To explore the relationship between fungal and bacterial enterotypes, we conducted a correlation analysis using the top 10 bacterial and fungal genera. We found a significant correlation between fungal and bacterial enterotypes (p < 0.05, Figure S6F). In the HAP group, the significantly enriched Bifidobacterium was positively correlated with the dominant fungal species Saccharomyces and Malassezia in the E3 enterotype. These findings indicate a significant correlation between fungal and bacterial communities. Further analysis of the relationships between archaea, bacteria, and fungal enterotypes using the top 10 genera for each group showed a significant correlation between archaea and bacterial enterotypes (p < 0.05, Figure S6G). The dominant species Escherichia in bacterial E2 enterotype showed a significant positive correlation with the dominant species Methanobrevibacter in archaea E5 enterotype. Moreover, our results revealed a significant correlation between archaea and fungal enterotypes (p < 0.05, Figure S6H). Notably, the archaea genus Thermococcus, significantly enriched in the HAP group, showed a significant positive correlation with the fungal genus Penicillium, which was also enriched in the same group (p < 0.05, Figure S6I). These findings provide strong evidence for significant correlations between archaea, fungi, and bacterial communities, with their mutual promotion likely manifesting through various mechanisms such as exchange of metabolic products, nutritional complementarity, co-metabolism, signaling, and biofilm formation. Through these processes, microbial communities can coexist and support each other’s growth in complex and extreme environmental conditions. In particular, in high-altitude environments, hypoxia and resource scarcity may foster more intimate interdependent relationships between these microbial communities, allowing them to jointly adapt to environmental pressures.

**Enterotypes for distinguishing populations from different regions using random forest classification**

Given the differences in enterotype compositions between the populations, we further investigated whether these enterotypes could distinguish between CAP and HAP. We evaluated the predictive ability of a binary classification model to differentiate between high-altitude adenoma and coastal adenoma populations. In the high-altitude adenoma group, E1 enterotype was dominant, and we used the top 10 dominant bacterial genera from E1 to construct a random forest model for classification. The classification AUC was 0.79 (F1 score = 0.78), with sensitivity and specificity of 0.69 and 0.73, respectively (Figure S7A). Similarly, the fungal enterotype and archaea enterotype were used to construct prediction models, with the fungal enterotype AUC of 0.64 (F1 score = 0.70), sensitivity and specificity of 0.55 and 0.64 (Figure S7B), and the archaea enterotype AUC of 0.68 (F1 score = 0.73), sensitivity and specificity of 0.57 and 0.68 (Figure S7C). To assess the performance of multi-domain models, we combined the top 10 bacterial genera from E1 and the top 10 fungal genera from E3, achieving an AUC of 0.82, outperforming the bacterial model alone (AUC = 0.79, Figure S7D). Similarly, combining the top 10 bacterial genera from E1 and the top 10 archaea genera from E5 resulted in an AUC of 0.79, surpassing the archaea model alone (AUC = 0.68, Figure S7E). Additionally, combining the top 10 bacterial genera from E1, the top 10 fungal genera from E3, and the top 10 archaea genera from E5 resulted in an AUC of 0.85, outperforming models using only bacterial, fungal, or archaea data (Figure S7F). These results emphasize the enhanced predictive ability of multi-domain models incorporating bacterial, fungal, and archaea biomarkers for distinguishing between high-altitude and coastal populations.
