# Supplementary material for: The study on the identification of cross-boundary microbiome enterotypes between high-altitude and coastal populations and their predictive value
Source: BMC Microbiol. 2026 Jan 29;26:225. doi: 10.1186/s12866-025-04578-0 (PMC12973879; doi:10.1186/s12866-025-04578-0)
Supplement: Supplementary file 9 — Supplementary Material 9. [file 12866_2025_4578_MOESM9_ESM.docx]

**SUPPLEMENTARY TEXT 2**

**Results**

**Overall characteristics of bacterial enterotype microbial communities in high-altitude control and high-altitude adenoma populations**

First, a PCoA plot was used to analyze the microbial communities in HCP and HAP (Figure S8A). Then, JSD distance metrics were applied for clustering 111 samples based on the relative abundance of bacterial genera at the genus level. K values of 2, 3, and 4 were selected based on profile width, CH index, DBI index, and Dunn index for clustering (Figure S1C). Based on the dominant genera in each group, two bacterial enterotypes were designated as Prevotella (E1, n=44) and Bacteroides (E2, n=67). Three fungal enterotypes were designated as Saccharomyces (E3, n=30), Malassezia (E4, n=16), and Aspergillus (E8, n=65). Four archaeal enterotypes were designated as Methanobrevibacter (E5, n=27), Methanosarcina (E6, n=25), Methanosphaera (E7, n=52), and Thermococcus (E9, n=7). The bacterial genera proportions in each enterotype are shown in Figure S8B. In summary, *Prevotella* (50.56%) and *Faecalibacterium* (6.98%) were relatively abundant in the E1 enterotype, while *Bacteroides* (22.49%) and *Phocaeicola* (17.55%) were relatively abundant in the E2 enterotype. *Saccharomyces* (79.75%) was abundant in the E3 enterotype, and *Malassezia* (48.24%) and *Candida* (13.47%) were abundant in the E4 enterotype. *Aspergillus* (9.5%) dominated in the E8 enterotype. *Methanobrevibacter* (90.57%) was abundant in the E5 enterotype, while *Methanosarcina* (22.56%) and *Methanosphaera* (21.35%) were abundant in the E6 enterotype. *Methanosphaera* (84.12%) was dominant in the E7 enterotype, and *Thermococcus* (84.85%) was predominant in the E9 enterotype. Clinical factors such as age and sex were also analyzed across enterotypes, and no significant statistical differences were found between them (P>0.05) (Figure S8D).

**Composition of gut microbiota at the genus level in E1 enterotype for two regional populations**

In the E1 enterotype, the bacterial composition at the genus level for two regional populations is shown in Figure S9A. In both populations, *Prevotella, Faecalibacterium, Bacteroides, Streptococcus,* and *Megamonas* were identified as the five dominant genera. The PCA plot shows no significant difference in the microbiota composition between the two populations (P>0.05) (Figure S9B). Further analysis using LDA revealed three genera with significant differences between HCP and HAP participants (Figure S9C). Among them, *Vibrio, Flavobacterium,* and *Acidovorax* were significantly enriched in the HCP group.

**Composition of gut microbiota at the genus level in E2 enterotype for two regional populations**

In the E2 enterotype, Figure S9D shows the relative abundance of bacterial genera for the two regional populations. In both populations, *Bacteroides, Phocaeicola, Faecalibacterium, Bifidobacterium,* and *Escherichia* were the five main bacterial genera. The PCA plot shows no significant separation between the two groups (P>0.05) (Figure S9E). Additionally, LDA identified three genera significantly enriched in the HCP group, including *Roseburia, Blautia,* and *Gemella*, while in the HAP group, *Klebsiella* and *Phyllobacterium* were more predominant. To validate the reliability of the enterotype classification, we analyzed all samples from high-altitude populations without classifying them by enterotype. The results revealed differences in 12 intestinal genera between the two groups (Figure S9G). In summary, four genera (*Klebsiella, Vibrio, Microbacterium,* and *Thiothrix*) were significantly enriched in the HAP group, while eight biomarkers (*Roseburia, Catenibacterium, Blautia*, etc.) had the highest abundance in the HCP group.

**Composition of gut microbiota at the genus level in E3 enterotype for two regional populations**

In the E3 enterotype, the fungal composition at the genus level for the two regional populations is shown in Figure S10A. In both populations, *Saccharomyces, Malassezia, Synchytrium, Candida,* and *Aspergillus* were the five dominant genera. Compared to the HCP group, the relative abundance of *Malassezia* and *Synchytrium* was higher in the HAP group, while the relative abundance of *Heterobasidion* was lowest in the HAP group, and it was significantly higher in the HCP group. The PCA plot shows no significant difference in microbiota composition between the two groups (P>0.05) (Figure S10B). Further analysis using LDA identified four genera with significant differences between the HCP and HAP groups (Figure S10C). Among them, *Malassezia, Kazachstania, Cladosporium,* and *Wallemia* were significantly enriched in the HAP group.

**Composition of gut microbiota at the genus level in E4 enterotype for two regional populations**

In the E4 enterotype, Figure S10D shows the relative abundance of fungal genera at the genus level for the two regional populations. In both populations, *Malassezia, Candida, Saccharomyces, Aspergillus,* and *Alternaria* were the five main fungal genera. Compared to the HCP group, the relative abundance of *Malassezia* and *Candida* was higher in the HAP group, while the relative abundance of *Pyricularia* was lowest in the HAP group, and it was significantly higher in the HCP group. The PCA plot shows no significant difference between the two groups (P>0.05) (Figure S10E). Additionally, LDA identified three genera significantly enriched in the HCP group, including *Aspergillus, Penicillium,* and *Clavispora*, while in the HAP group, three genera were identified as dominant, including *Komagataella, Blumeria,* and *Phanerochaete.*

**Composition of gut microbiota at the genus level in two regional populations within the E5 enterotype**

In the E5 enterotype, the archaeal genus composition of the two regional populations is shown in Figure S11A. *Methanobrevibacter, Methanosphaera, Thermococcus, Sulfolobus,* and *Methanosarcina* were identified as the five dominant genera in both populations. PCA analysis indicates no significant difference in the microbiota composition between the two regional populations (P=0.106) (Figure S11B). Further analysis using LDA to identify species-level differences between the two groups revealed one genus with a difference between HCP and HAP (Figure S11C), with *Thermococcus* being significantly enriched in the HCP group.

**Composition of the gut microbiota at the genus level in two regional populations of the E6 enterotype**

In the E6 enterotype, Figure S11D shows the relative abundance of archaeal genera in the two regional populations. In both populations, *Methanosarcina, Methanosphaera, Methanobrevibacter, Thermococcus,* and *Sulfolobus* were the five major archaeal genera. The PCA plot shows no significant separation between the two groups (P=0.671) (Figure S11E).

**Composition of the gut microbiota at the genus level in two regional populations of the E7 enterotype**

In the E7 enterotype, Figure S11F shows the relative abundance of archaeal genera in the two regional populations. In both populations, *Methanosphaera, Thermococcus, Methanobrevibacter, Sulfolobus,* and *Methanosarcina* were the five major archaeal genera. The PCA plot shows no significant separation between the two groups (P=0.862) (Figure S11G).

**Composition of the gut microbiota at the genus level in two human populations of the E8 enterotype**

Additionally, the composition of the E8 enterotype microbiota at the genus level was analyzed to describe the specific changes in the gut microbiota of different regional populations (Figure S10G). In each population, *Aspergillus, Yarrowia, Saccharomyces, Malassezia,* and *Penicillium* were identified as dominant genera. Compared to the HCP group, we observed higher relative abundances of *Malassezia* and *Penicillium* in the HAP group, while the relative abundance of MuCor was lowest in the HAP group, and significantly higher in the HCP group. Based on the PCA plot, no obvious structural difference in the microbiota was observed between the two groups (Figure S10H). LDA showed that the three groups shared eight dominant genera, with seven detected in the HCP group and one in the HAP group (Figure 25C). Among them, the HCP group was dominated by *Lasiodiplodia, Spathaspora,* and *Pyricularia*, while the HAP group was dominated by *Exophiala* (Figure S10I). To validate the reliability of the three enterotype classification criteria, we also analyzed samples from different regional populations without enterotype classification. The results showed differences in six genera of gut microbiota between the two groups (Figure S10J). In summary, three fungal genera (including Y*arrowia, Spathaspora,* and *Torulaspora*) were significantly enriched in the HCP group, while three biomarkers (including *Blumeria, Melampsora,* and *Phanerochaete*) had the highest abundance in the HAP group.

**Composition of the gut microbiota at the genus level in two human populations of the E9 enterotype**

Additionally, the composition of the E9 enterotype microbiota at the genus level was analyzed to describe the specific changes in the gut microbiota of different regional populations (S11H). In each population, *Thermococcus, Methanobrevibacter, Pyrobaculum, Methanosphaera,* and *Natronorubrum* were identified as dominant genera. Compared to the HCP group, we observed a higher relative abundance of *Methanosphaera* in the HAP group, while the relative abundance of *Halomicroarcula* was lowest in the HAP group. Based on the PCA plot, no obvious structural difference in the microbiota was observed between the two groups (Figure S11I). To validate the reliability of the three enterotype classification criteria, we also analyzed samples from different regional populations without enterotype classification. The results showed differences in one genus of gut microbiota between the two groups (Figure S11J). In summary, one archaeal genus (*Methanomethylovorans*) was significantly enriched in the HAP group.

**Functional differences in gut microbiota across enterotypes**

To characterize the biological activity potential of bacterial enterotypes and their potential metabolic mechanisms, we utilized the MetaCyc gene functional annotation database for cross-domain microbiome genomic predictions of gut microbiota metabolic functions in the two populations (Figure S12A). In the E1 enterotype, metabolic pathways related to ASPASN-PWY and PWY-5103 (P < 0.05) may influence the energy balance and protein synthesis capacity of high-altitude populations in hypoxic environments. Amino acid metabolism is crucial for immune function, protein synthesis, and muscle repair (Figure S12A). Additionally, excessive expression of pathways related to fructan synthesis (PWY-822, P < 0.05) was observed in the E1 enterotype, suggesting that fructans play an important role in plant stress resistance and energy storage, and may also help maintain gut health by promoting the growth of probiotics. The dominant genera Prevotella and Faecalibacterium in the E1 enterotype were also significantly associated with ASPASN-PWY (Figure S12B). In the E3 enterotype, pathways closely related to P101-PWY, which are primarily associated with ectoine biosynthesis, were observed. Ectoine is a natural compound with protective and stress-resistant effects, typically found in microorganisms from extreme environments. In the high-altitude environment, due to low oxygen content, microorganisms and the microbiota of high-altitude populations may increase their stress resistance through ectoine synthesis, helping them adapt to the hypoxic environment and maintain stable cell function. Furthermore, the E4 enterotype was mainly associated with the degradation of leucine (LEU-DEG2-PWY). L-leucine, one of the branched-chain amino acids, is involved in protein synthesis, energy metabolism, and cell signaling. In the high-altitude hypoxic environment, regulating leucine metabolism may help improve muscle energy metabolism efficiency, adapt to prolonged physical activity, and cope with high physiological loads by optimizing the degradation pathway of leucine, especially in hypoxic conditions (Figure S12C). Additionally, the dominant fungal genus Malassezia in the E3 enterotype was closely related to the P101-PWY pathway (Figure S12D). In the E5 enterotype, metabolic pathways related to the synthesis of furanosylglucuronic acid (PWY-7312, P < 0.05) were closely associated. PWY-7312 (dTDP-β-D-furanosylglucuronic acid synthesis) plays a key role in cell wall synthesis, immune evasion, and environmental adaptation in both archaea and bacteria (Figure S12E). In high-altitude populations, archaea may synthesize sugar antigens through this pathway to help them adapt to the extreme environment, and these metabolic products might interact with the host's immune system, improving the host's adaptation to extreme conditions. Additionally, the dominant genus Methanosphaera in the E5 enterotype was significantly positively correlated with the PWY-7312 pathway (Figure S12F). This may be related to the influence of low oxygen on the microbiota composition in high-altitude populations, as hypoxia may alter the metabolic patterns of the gut microbiota, leading to differences in energy acquisition and metabolic products compared to other populations. Furthermore, to explore the relationship between fungal and bacterial enterotypes, we performed a correlation analysis using the top 10 genera from both fungal and bacterial enterotypes. We observed significant correlations between fungal and bacterial enterotypes (p < 0.05, S12G). The dominant genus Megamonas in the bacterial E1 enterotype showed a significant positive correlation with Malassezia, the dominant genus in the fungal E4 enterotype, suggesting a significant relationship between fungal and bacterial communities. Additionally, to explore the relationship between archaea, bacteria, and fungal enterotypes, we performed a correlation analysis using the top 10 genera from archaea, bacteria, and fungal enterotypes. We observed significant correlations between the archaea and bacteria enterotypes (p < 0.05, S12H). The dominant genus Faecalibacterium in the bacterial E1 enterotype was significantly positively correlated with Methanosphaera, the dominant genus in the archaeal E7 enterotype. Furthermore, our results showed a significant correlation between archaea and fungal enterotypes (p < 0.05, S12I). The dominant genus Malassezia in the fungal E3 enterotype was significantly positively correlated with Natronorubrum, the dominant genus in the archaeal E9 enterotype, providing evidence of a significant correlation between archaea, bacteria, and fungal communities.
